# Base excision repair of oxidative DNA damage coupled with removal of a CAG repeat hairpin attenuates trinucleotide repeat expansion

**DOI:** 10.1093/nar/gkt1372

**Published:** 2014-01-14

**Authors:** Meng Xu, Yanhao Lai, Justin Torner, Yanbin Zhang, Zunzhen Zhang, Yuan Liu

**Affiliations:** ^1^Department of Chemistry and Biochemistry, Florida International University, Miami, FL 33199, USA, ^2^Department of Environmental Health, West China School of Public Health, Sichuan University, Chengdu, Sichuan 610041, P. R. China and ^3^Department of Biochemistry and Molecular Biology, Miller School of Medicine, University of Miami, Miami, FL 33136, USA

## Abstract

Trinucleotide repeat (TNR) expansion is responsible for numerous human neurodegenerative diseases. However, the underlying mechanisms remain unclear. Recent studies have shown that DNA base excision repair (BER) can mediate TNR expansion and deletion by removing base lesions in different locations of a TNR tract, indicating that BER can promote or prevent TNR expansion in a damage location–dependent manner. In this study, we provide the first evidence that the repair of a DNA base lesion located in the loop region of a CAG repeat hairpin can remove the hairpin, attenuating repeat expansion. We found that an 8-oxoguanine located in the loop region of CAG hairpins of varying sizes was removed by OGG1 leaving an abasic site that was subsequently 5′-incised by AP endonuclease 1, introducing a single-strand breakage in the hairpin loop. This converted the hairpin into a double-flap intermediate with a 5′- and 3′-flap that was cleaved by flap endonuclease 1 and a 3′-5′ endonuclease Mus81/Eme1, resulting in complete or partial removal of the CAG hairpin. This further resulted in prevention and attenuation of repeat expansion. Our results demonstrate that TNR expansion can be prevented via BER in hairpin loops that is coupled with the removal of TNR hairpins.

## INTRODUCTION

Trinucleotide repeat (TNR) expansion is associated with >40 neurodegenerative diseases such as Huntington’s disease (HD) [(CAG)_n_/(CTG)_n_], myotonic dystrophy [(CTG)_n_/(CAG)_n_], fragile X syndrome [(CGG)_n_/(CCG)_n_] and Friedreich’s ataxia [(GAA)_n_/(TTC)_n_] (1–3). The molecular basis underlying TNR expansion is proposed to be the formation of non-B form DNA secondary structures during DNA replication, repair and recombination, including hairpins, tetraplexes and triplexes (4–9). These structures can result in a series of effects that ultimately lead to TNR expansion. These include DNA polymerase stalling, which further causes DNA slippage and misalignment (9,10), replication fork stalling (8,11), inhibition of cleavage of hairpins by flap endonuclease 1 (FEN1) (12–14), trapping of mismatch repair proteins (15,16) and disruption of the coordination among repair enzymes (17). Thus, TNR expansion is a consequence of genome integration of unresolved DNA secondary structures formed during DNA replication, repair and recombination.

TNRs contain a high proportion of guanines, which are susceptible to loss of electrons and oxidation (18). This causes TNRs to be hotspots of oxidative DNA damage. Repair of oxidative DNA damage in the context of TNR tracts was previously shown to be associated with repeat expansion and deletion (19–21). An increased level of 8-oxoguanine (8-oxoG) was shown to be correlated with age-dependent CAG repeat expansions in the brain of HD transgenic mouse models (19,22). The oxidative DNA damage from H_2_O_2_ caused small CAG repeat expansions in human cells (19,21), and the damage from potassium bromate increased the levels of 8-oxoG and CGG repeat expansions in the germ cells of fragile X syndrome premutation mice (23). H_2_O_2_ can also increase TNR deletions in bacteria and mouse kidney cells (24,25). We recently discovered that base excision repair (BER) of an abasic lesion can lead to CAG repeat deletion by inducing the formation of a template hairpin, promoting the coordination between DNA polymerase β (pol β) hairpin bypass and FEN1 alternate flap cleavage (20). Furthermore, we have found that an abasic lesion located at different positions of a CTG repeat tract can result in either repeat expansion or deletion (21). Thus, oxidative DNA damage and BER play dual roles in modulating TNR instability.

Unpaired bases were found to be 10-fold more accessible to oxidative DNA damaging agents than paired bases in duplex DNA (26). Guanines located at the loop region of a TNR hairpin are hypersensitive to oxidative DNA damaging agents such as peroxynitrite (27). Moreover the oxidized base lesion 8-oxoG located in the stem region of a CAG repeat hairpin is preferentially relocated to the loop region via a dynamic intrastrand rearrangement of the repeats (28). However, it was shown that a base lesion in a hairpin loop that was detached from a CAG repeat tract was refractory to cleavage by 8-oxoG DNA glycosylase (OGG1) (29). This results in the accumulation of oxidative DNA damage in TNR hairpin loops and persistence of hairpin structures, leading to multiple rounds of ‘toxic oxidation cycles’ and causing TNR expansion (3,29). Thus, removal of an oxidized base lesion in the loop region of a TNR hairpin appears to be a critical step for preventing toxic oxidation cycles and TNR expansion. However, it is unknown how a base lesion in a TNR hairpin can be removed for prevention of TNR expansion.

In this study, we provide the first evidence that an 8-oxoG in the loop of a TNR hairpin in a CAG repeat tract can be simultaneously removed with the hairpin via BER. This represents a novel pathway for preventing TNR expansion through BER coupled with removal of TNR hairpins.

## MATERIALS AND METHODS

### Materials

DNA oligonucleotides containing an 8-oxoG were synthesized by Eurofins MWG Operon (Huntsville, AL, USA). All other oligonucleotides were from Integrated DNA Technologies (IDT, Coralville, IA, USA). Deoxynucleoside 5′-triphosphates (dNTPs) and terminal deoxynucleotidyl transferase were from Fermentas (Glen Burnie, MD, USA). T4 polynucleotide kinase was from USB Corp. (Cleveland, OH, USA). All standard chemical reagents were from Sigma-Aldrich (St. Louis, MO, USA) and Thermo Fisher Scientific (Pittsburgh, PA, USA). The radionucleotides [γ-^32^P] ATP (6000 mCi/mmol) and Cordycepin 5′-triphosphate 3′-[α-^32^P] (5000 mCi/mmol) were purchased from PerkinElmer Inc. (Boston, MA, USA). Micro Bio-Spin 6 chromatography columns were from Bio-Rad (Hercules, CA, USA). The Mus81/Eme1 expression vector pET21d-Mus81/HIS-Eme1 was a generous gift from Dr Stephen West at Clare Hall Laboratories, London Research Institute, Cancer Research UK, Hertfordshire, UK. Purified OGG1, AP endonuclease 1 (APE1), pol β and DNA ligase I (LIG I) were generous gifts from Dr Samuel Wilson at the National Institute of Environmental Health Sciences, National Institutes of Health, Research Triangle Park, NC, or were expressed and purified according to the procedures described previously (30).

### Oligonucleotide substrates

Substrates containing a (CAG)_7_ or (CAG)_14_ hairpin with an 8-oxoG or a tetrahydrofuran (THF), an abasic site analog (used to represent an oxidized sugar in this study), located in the hairpin loop region were designed to mimic a TNR hairpin with a base lesion in the hairpin loop region. Substrates were constructed by annealing the damaged strands that contained (CAG)_13_ or (CAG)_20_ repeats to their template strand containing (CTG)_7_ repeats at a molar ratio of 1:1.5. Substrates with a 3′- and 5′-flap were constructed to mimic double-flap intermediates generated by APE1 5′-incision of an abasic site in the loop region of CAG repeat hairpins of varying sizes. Substrates were constructed by annealing an upstream primer containing a (CAG)_4_ or a (CAG)_7_ flap/hairpin, and a downstream primer containing a 5′-phosphorylated THF residue attached to a (CAG)_3_ or a (CAG)_7_ flap/hairpin with the template strand containing (CTG)_7_ repeats at a molar ratio of 1:1:1.5. For all the substrates, three CAG repeats flanked both the 5′- and 3′-side of the hairpins or flaps and base paired with three CTG repeats on the template strand. Substrates were radiolabeled at the 3′- or 5′-end of the damaged strand, the upstream primer or the downstream primer for measuring various types of enzymatic activities. The sequences of oligonucleotide substrates are listed in Supplementary Table S1. DNA size markers were synthesized by IDT.

### Protein expression and purification

FEN1 and Mus81/Eme1 were expressed in *E. coli* according to the procedures described previously (30,31). Briefly, cell pellets were lysed by a French press in lysis buffer containing 50 mM 4-(2-hydroxyethyl)-1-piperazineethanesulfonic acid (HEPES), pH 7.5, 30 mM NaCl, 1 mM dithiothreitol (DTT), 1 mM EDTA, 1 mM phenylmethylsulfonyl fluoride (PMSF), 1 mM benzamidine, 1 µg/ml pepstatin A and 1 µg/ml leupeptin. Cell lysates were then subjected to centrifugation at 12 000 rpm at 4°C for 30 min to separate soluble proteins from cell debris. For FEN1 purification, the supernatant from cell lysates was initially subjected to purification by a 10-ml Sepharose Q column operated by the AKTA Fast Protein Liquid Chromatography system (FPLC; GE Healthcare, Piscataway, NJ, USA). The flow-through was collected and dialyzed into buffer containing 50 mM HEPES, pH 7.5, 30 mM NaCl and 1 mM PMSF, and subsequently loaded onto a 5-ml CM column (Bio-Rad, Hercules, CA, USA). Fractions were obtained with elution by a linear gradient of NaCl ranging from 30 mM to 2 M, and peak fractions were combined and dialyzed into buffer containing 50 mM HEPES, pH 7.5, 30 mM NaCl and 1 mM PMSF and loaded onto a 1-ml Mono-S column (GE Healthcare, Piscataway, NJ, USA). Peak fractions were obtained by elution with a linear gradient of NaCl from 30 mM to 2 M. The activity of purified FEN1 was measured by monitoring FEN1 cleavage of a 3-nt flap that was radiolabeled at the 5′-end of the flap. Purified FEN1 was aliquoted and frozen at −80°C until further use.

Mus81/Eme1 heterodimer was expressed in the *E. coli* BL21(DE3)-RP strain (Agilent Technologies, Santa Clara, CA, USA) transformed with the expression vector pET21d-Mus81/HIS-Eme1. Two hundred milliliters of lysogeny broth (LB) medium with 50 µg/ml ampicillin was inoculated with a newly transformed single colony and shaken at 225 rpm at 37°C overnight. The overnight culture was then inoculated into three 1-liter LB cultures using 60 ml of overnight culture per liter. The newly inoculated culture was incubated at 30°C with shaking until absorbance at 600 nm reached ∼1.0. Subsequently, the expression of Mus81/Eme1 was induced by 10 µM of isopropyl β-D-1-thiogalactopyranoside (IPTG) at 30°C for 3 h. Cells were harvested by centrifugation at 3000 rpm and 4°C, for 40 min. Cell pellets were resuspended in lysis buffer containing 50 mM sodium phosphate, pH 7.0, 0.01% NP-40, 10% glycerol, 0.1 mM NaCl, 1 mM PMSF, 1 mM benzamidine, 1 µg/ml pepstatin A and 1 µg/ml leupeptin, and lysed by a French press. The cell lysates were centrifuged at 12 000 rpm, and 4°C, for 30 min. Supernatant was loaded onto a 10-ml P11 phospho-cellulose column (GE Healthcare, Piscataway, NJ, USA) using the AKTA FPLC system, and proteins were eluted with a linear gradient of NaCl ranging from 0.1 to 1 M. Peak fractions were collected and subjected to purification by Dynabeads coated with cobalt (Novex/Life Technologies Corp., Grand Island, NY, USA). Fractions (350 µl) were incubated with 50 µl of beads and 350 µl of binding buffer containing 50 mM sodium phosphate, pH 8.0, 300 mM NaCl and 0.001% Tween-20, and were rotated at 4°C for 2 h. Protein-bead complexes were pelleted via a magnet at 4°C for 2 min. Pellets were then washed with 300 µl of buffer containing 50 mM imidazole, three times. Mus81/Eme1 protein complexes were eluted with 100 µl of buffer containing 300 mM imidazole. The activity of Mus81/Eme1 was determined by measuring its cleavage of a 9 nt-3′-flap substrate that was radiolabeled at the 5′-end of the 3′-flap. Purified Mus81/Eme1 was aliquoted and frozen at −80°C until further use.

### *In Vitro* BER reconstituted with purified enzymes

*In vitro* BER of an 8-oxoG or oxidized abasic site (THF) in the loop region of CAG repeat hairpins with varying sizes was performed by incubating 25 nM substrates containing a (CAG)_7_ or (CAG)_14_ hairpin with an 8-oxoG or a THF residue in the hairpin loop with indicated concentrations of OGG1, APE1, pol β, Mus81/Eme1, FEN1 and LIG I. BER of single-strand DNA (ssDNA) break intermediates resulting from APE1 5′-incision of an abasic site in the hairpin loops were reconstituted by incubating purified Mus81/Eme1, pol β, FEN1 and LIG I with 25 nM substrates that contained different sizes of 3′- and 5′-flaps. Reactions were performed with BER reaction buffer containing 50 mM Tris–HCl, pH 7.5, 50 mM KCl, 0.1 mM EDTA, 0.1 mg/ml bovine serum albumin, 0.2 mM DTT and 0.01% Nonidet P-40 with 5 mM MgCl_2_, 2 mM ATP and 50 μM dNTPs. Reaction mixtures were incubated at 37°C for 30 min, and terminated by incubation at 95°C for 10 min in stopping buffer containing 95% formamide and 10 mM EDTA. Substrates and products were separated by 15% urea-denaturing polyacrylamide gel electrophoresis and detected by a Pharos FX Plus PhosphorImager from Bio-Rad. Substrates were ^32^P-labeled at the 3′- or 5′-end of the strand with an 8-oxoG or a THF residue, the upstream primer and downstream primer.

### Probing of hairpin structures and 3′- and 5′-flaps by Mung Bean Nuclease and S1 Nuclease digestion

Formation of CAG repeat hairpins in hairpin-containing substrates or the 3′- and 5′-flaps in double-flap substrates was probed using Mung Bean Nuclease according to the procedure described by Xu *et al.* (20). Briefly, substrates (200 nM) were incubated with 0.1 or 0.15 U Mung Bean Nuclease in its reaction buffer at 37°C for 1, 2, 3 and 5 min. Formation of CAG repeat hairpins in the substrates was also probed using 0.2 or 0.5 U S1 Nuclease and 200 nM substrates. Reactions were assembled in S1 Nuclease reaction buffer containing 50 mM sodium acetate (pH 4.5), 280 mM NaCl and 4.5 mM ZnSO_4_ and incubated at 37°C for 3, 5 and 10 min. Reaction mixtures were subsequently subjected to protease K digestion at 55°C for 30 min to remove the nucleases. Substrates and products were separated by 15% urea-denaturing polyacrylamide gel electrophoresis and detected by a PhosphorImager.

## RESULTS

### A DNA base lesion located in a CAG repeat hairpin loop can be removed by OGG1 and APE1

**Figure 1.**
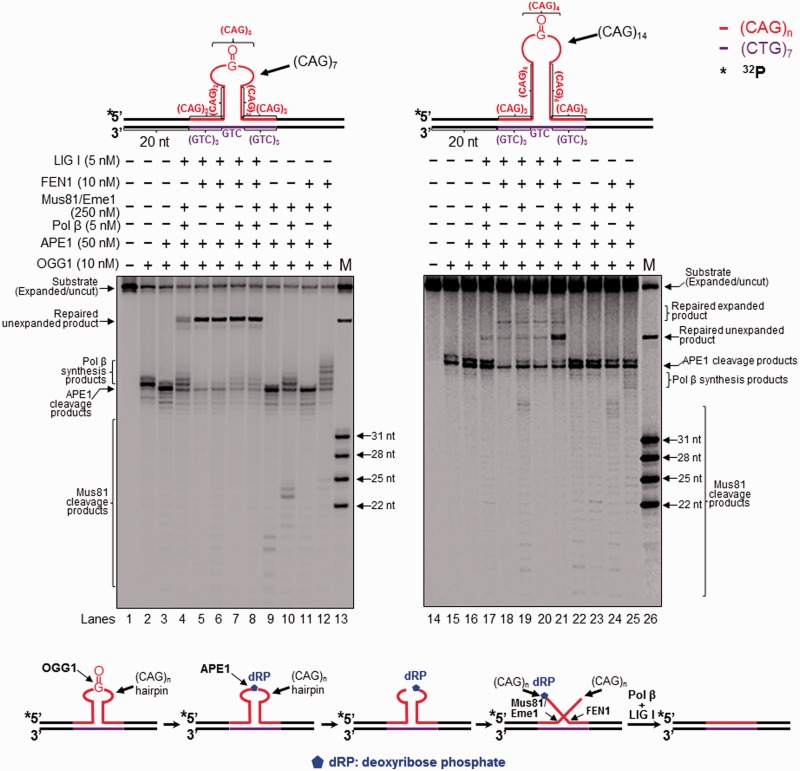
Attenuation of CAG repeat expansion through repair of 8-oxoG in the hairpin loop and Mus81/Eme1 cleavage. Repair of an 8-oxoG in the loop region of a CAG hairpin and Mus81/Eme1 cleavage activity were examined with substrates containing a (CAG)_7_ (left panel) or (CAG)_14_ hairpin (right panel) with an 8-oxoG in the hairpin loop region. Lanes 1 and 14 correspond to substrates only. Lanes 2 and 15 correspond to reaction mixtures with 10 nM OGG1. Lanes 3 and 16 correspond to reaction mixtures with 10 nM OGG1 and 50 nM APE1. Lanes 4 and 17 correspond to reaction mixtures with 250 nM Mus81/Eme1, 5 nM pol β, 10 nM OGG1, 50 nM APE1 and 5 nM LIG I in the absence of FEN1. Lanes 5–6 and lanes 18–19 correspond to reaction mixtures with 10 nM OGG1, 50 nM APE1, 10 nM FEN1 and 5 nM LIG I in the absence or presence of 250 nM Mus81/Eme1. Lanes 7–8 and lanes 20–21 correspond to reaction mixtures with 10 nM OGG1, 50 nM APE1, 5 nM pol β, 10 nM FEN1, 5 nM LIG I in the absence or presence of 250 nM Mus81/Eme1. Lanes 9–10 and 22–23 correspond to reaction mixtures with 10 nM OGG1, 50 nM APE1, 250 nM Mus81/Eme1 in the absence and presence of 5 nM pol β. Lanes 11–12 and lanes 24–25 represent reaction mixtures with 10 nM OGG1, 50 nM APE1, 250 nM Mus81/Eme1 and 5 nM FEN1 in the absence and presence of pol β. Lanes 13 and 26 correspond to a series of synthesized size markers (M) for illustrating the size of repaired products and Mus81/Eme1 cleavage products. Substrates were ^32^P-labeled at the 5′-end of the hairpin-containing strand (expanded/uncut strand). Substrates are illustrated schematically above the gels. A scheme that indicates removal of a CAG repeat hairpin with an 8-oxoG is illustrated below the gels. The sizes of Mus81/Eme1 cleavage products are illustrated in nucleotides.

**Figure 2.**
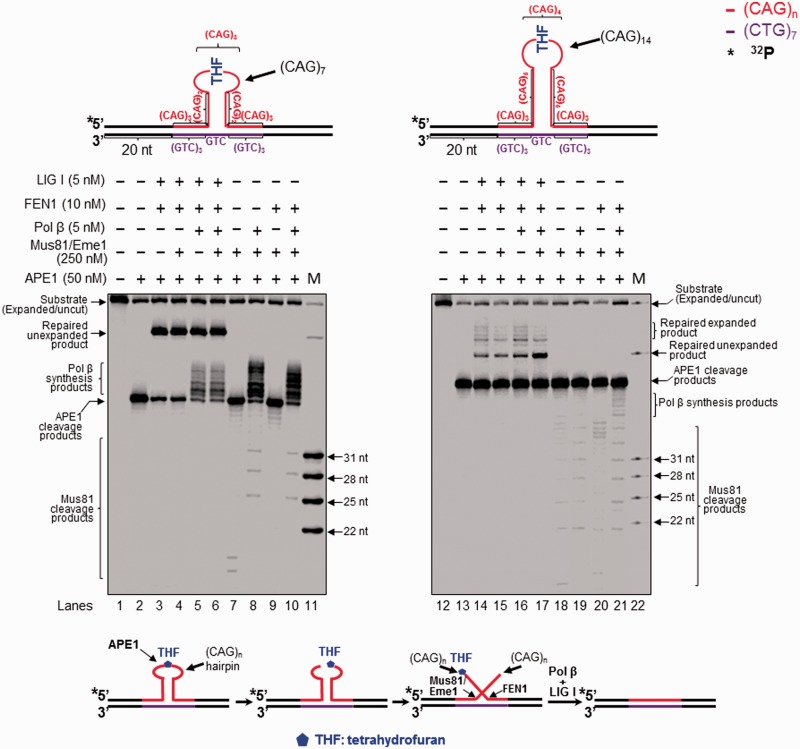
Attenuation of CAG repeat expansion through repair of an abasic site in the hairpin loop and Mus81/Eme1 cleavage. Repair of an abasic site in the loop region of a CAG hairpin and Mus81/Eme1 cleavage were examined with substrates containing a (CAG)_7_ (left panel) or (CAG)_14_ hairpin (right panel) with a THF in the hairpin loop region. Lanes 1 and 12 correspond to substrates only. Lanes 2 and 13 represent reaction mixtures with 50 nM APE1. Lanes 3, 5, 14 and 16 represent reaction mixtures with or without 5 nM pol β in the presence of 50 nM APE1, 10 nM FEN1 and 5 nM LIG I. Lanes 4, 6, 15 and 17 correspond to reaction mixtures with or without 5 nM pol β in the presence of 50 nM APE1, 5 nM FEN1, 250 nM Mus81/Eme1 and 5 nM LIG I. Lanes 7–8 and 18–19 correspond to reaction mixtures with or without 5 nM pol β in the presence of 250 nM Mus81/Eme1 and 50 nM APE1. Lanes 9–10 and 20–21 represent reaction mixtures with or without 5 nM pol β in the presence of 10 nM FEN1, 250 nM Mus81 and 50 nM APE1. Lanes 11 and 22 represent synthesized size markers (M). Substrates were ^32^P-labeled at the 5′-end of their damaged strands and are illustrated schematically above the gels. A scheme that indicates removal of a CAG repeat hairpin with a THF residue is illustrated below the gels. The size of Mus81/Eme1 cleavage products is illustrated in nucleotides.

OGG1 is a key enzyme that initiates BER in mammalian cells by removing an oxidized base lesion, 8-oxoG, leaving an abasic site that is incised by APE1 (32). The enzyme removes an 8-oxoG in a CAG hairpin loop that is detached from duplex DNA 700-fold slower than it does in duplex DNA (29). To determine if OGG1 can remove an 8-oxoG in a CAG hairpin loop that is located in duplex DNA, we examined OGG1 cleavage of an 8-oxoG in the loop region of a (CAG)_7_ or (CAG)_14_ hairpin in a CAG repeat tract. To verify the presence of an 8-oxoG and abasic site (THF residue) in the loop region of the substrates, we initially probed the formation of hairpins in the CAG repeat tract of the substrates using ssDNA-specific nucleases, Mung Bean Nuclease and S1 Nuclease. We found that Mung Bean Nuclease cleavage on the (CAG)_7_-8-oxoG hairpin substrate mainly resulted in products with 36, 39, 42 and 45 nt (Supplementary Figure S1A, lanes 2–4), whereas its cleavage on the (CAG)_7_-THF substrate resulted in products with 35, 39, 41 and 44 nt (Supplementary Figure S2A, lanes 2–4). Consistent with the results of Mung Bean Nuclease cleavage, S1 Nuclease cleavage on the (CAG)_7_-8-oxoG and (CAG)_7_-THF hairpin substrates resulted in products with 38, 39, 41, 42, 43 and 44 nt (Supplementary Figure S1B and S2B, lanes 2–4). The results indicate the formation of a small hairpin that contained a single-strand (CAG)_3_ loop with an 8-oxoG or THF and a (CAG)_4_ stem (Supplementary Figures S1A and S2A) in the duplex of the substrates. Mung Bean Nuclease cleavage on the (CAG)_14_-8-oxoG substrate mainly resulted in products with 45, 49, 52, 55 and 58 nt (Supplementary Figure S1A, lanes 7–9). For the (CAG)_14_-THF hairpin substrate, the nuclease cleavage led to production of products with 45, 48 and 52 nt (Supplementary Figure S2A, lanes 7–9), whereas S1 Nuclease cleavage on the (CAG)_14_-8-oxoG and (CAG)_14_-THF hairpin substrates resulted in products with 45, 49, 51 and 54 nt (Supplementary Figure S1B and S2B, lanes 7–9). This indicates that the substrates contained a hairpin with a (CAG)_4_ loop with an 8-oxoG or THF residue and 10 CAG repeats in the stem region. Thus, our results demonstrate that the 8-oxoG and THF residue (abasic site) were located in the loop region rather than in the stem of the hairpin substrates. This is consistent with a finding showing that a DNA base lesion such as an 8-oxoG is preferentially located in the loop region of a hairpin (28). We then determined the activities of OGG1 and APE1 on these substrates and discovered that ∼70% of 8-oxoG in the loop region of a small (CAG)_7_ hairpin was removed (Figure 1, lane 2), leaving an abasic site that was efficiently cleaved by APE1 (Figure 1, lane 3; Figure 2, lane 2). About 40% of 8-oxoG in the loop region of a large (CAG)_14_ hairpin was removed (Figure 1, lane 15), and the resulting abasic site was efficiently incised by APE1 (Figure 1, lane 16; Figure 2, lane 13). These results indicate that OGG1 and APE1 can remove an 8-oxoG and abasic site in the loop region of a hairpin located in a CAG repeat tract, initiating BER. This further indicates that OGG1 and APE1 cleavage activity on an 8-oxoG and abasic site is sufficient to initiate BER in a CAG hairpin loop region, although their activities decrease as the size of the hairpin increases.

### BER of a base lesion in the loop region of a CAG repeat hairpin resulted in removal of the hairpin

To determine if removal of an 8-oxoG and an abasic lesion located at a CAG repeat hairpin may result in any repaired product, we reconstituted BER for removing a base lesion in the loop region of a (CAG)_7_ or (CAG)_14_ hairpin substrate. Surprisingly, we found that BER in the small (CAG)_7_ hairpin loop generated a product with the same length as the template strand. We designated this product as ‘unexpanded product’ (Figure 1, lanes 4–8; Figure 2, lanes 3–6). BER in the large (CAG)_14_ hairpin loop resulted in the unexpanded product as well as the products that were shorter than the hairpin-containing substrate strand, but longer than the template strand. We named these products ‘expanded products’ (Figure 1, lanes 18–21; Figure 2, lanes 14–17). The results indicate that BER of a base lesion in the loop region of a CAG repeat hairpin removed the entire small (CAG)_7_ and large (CAG)_14_ hairpin, but also removed a part of a large (CAG)_14_ hairpin. Interestingly, we found that production of the repair products required the presence of either a 3′-5′ endonuclease such as Mus81/Eme1, which was used to demonstrate the role of a 3′-flap endonuclease in removing a TNR hairpin, or FEN1 (Figure 1, lanes 4–5 and lanes 17–18) in BER reactions because repair reactions without flap endonucleases failed to convert the APE1 product into the repaired product even when base lesion repair was forced to short-patch BER (Supplementary Figure S3, lanes 5 and 10). This suggests that APE1 resulted in a 5′-incised hairpin intermediate and flap intermediates that subsequently prevented DNA synthesis by pol β and DNA ligation (Supplementary Figure S3, the scheme below the gels). Because BER enzymes cannot directly remove the APE1 incised hairpin, this further suggests that BER in the context of a hairpin loop is accomplished through conversion of a hairpin stem into a double-flap intermediate. Thus, we suggest that incision of a hairpin loop by APE1 converted some of the hairpin stems into double-flap intermediates that can be subsequently processed by Mus81/Eme1 and FEN1. This further leads to removal of the hairpin and ultimately production of the repaired unexpanded product and shortened expansion products. Furthermore, we found that for 8-oxoG-containing hairpin substrates, the unexpanded product was generated in the presence of pol β and Mus81/Eme1 without the need of FEN1 (Figure 1, lanes 4 and 17). For THF-containing substrates, FEN1 was required for production of all repaired products (Figure 2, lanes 3–6, and lanes 14–17). This is because a THF residue that mimics an oxidized sugar, cannot be removed through β-elimination by pol β dRP lyase via short-patch BER. Thus it has to be removed by long-patch BER through FEN1 cleavage of the nucleotide attached to the residue (32). Our results demonstrate that both a 3′-5′ endonuclease and FEN1 play an important role in removing a hairpin during BER of a base lesion in a hairpin loop presumably by cleaving a 3′-flap or a 5′-flap.

### The 3′-5′ endonuclease Mus81/Eme1 removed the entirety of a CAG repeat hairpin by cleaving an upstream 3′- flap during BER, preventing CAG repeat expansion

**Figure 3.**
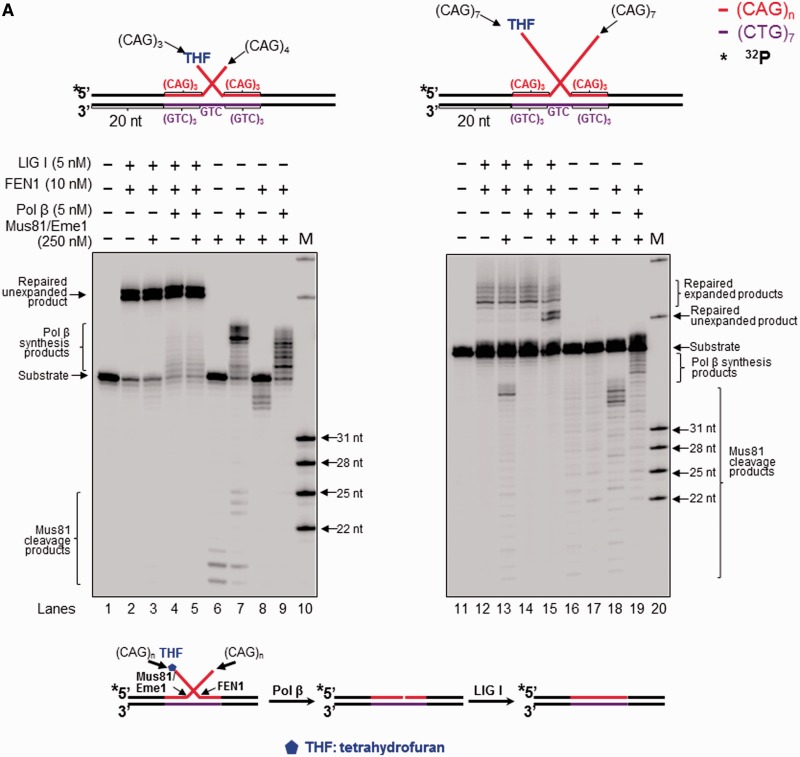

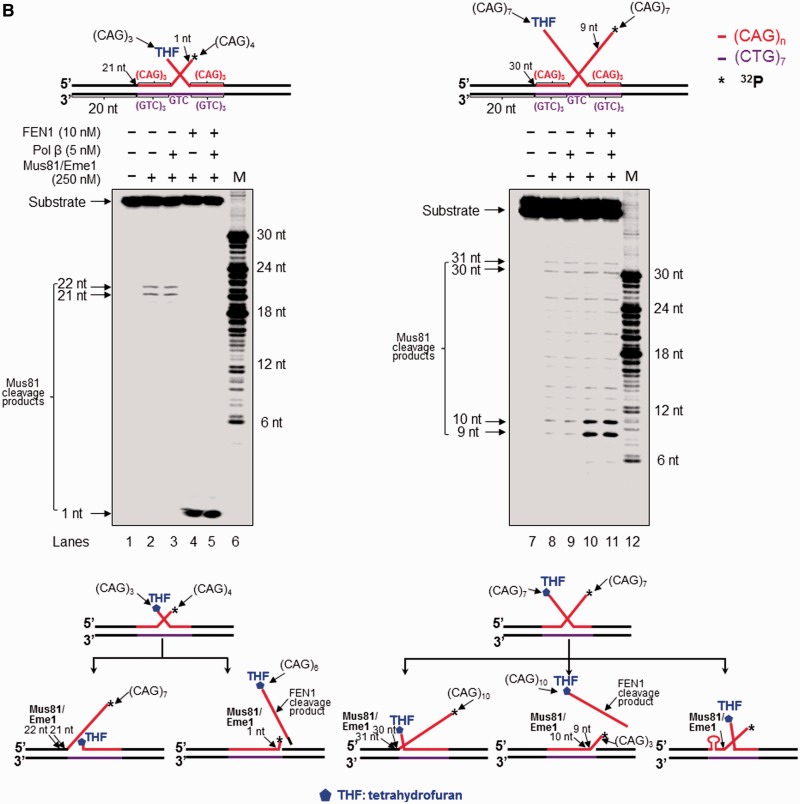
(**A**) Processing of a CAG repeat double-flap intermediate during BER prevented or attenuated CAG repeat expansion. Processing of a CAG repeat double-flap substrate and its effects on repeat stability during BER was examined with substrates containing an upstream 3′-(CAG)_4_ and a downstream 5′-THF-(CAG)_3_ (left panel) or an upstream 3′-(CAG)_7_ flap and a downstream 5′-THF-(CAG)_7_ flap (right panel). Lanes 1 and 11 represent substrates only. Lanes 2, 4, 12 and 14 correspond to reaction mixtures with or without 5 nM pol β in the presence of 10 nM FEN1 and 5 nM LIG I. Lanes 3, 5, 13 and 15 correspond reaction mixtures with or without 5 nM pol β in the presence of 250 nM Mus81/Eme1, 10 nM FEN1 and 5 nM LIG I. Lanes 6–7 and 16–17 represent reaction mixtures with or without 5 nM pol β in the presence of 250 nM Mus81/Eme1. Lanes 8–9 and 18–19 correspond to reaction mixtures with or without 5 nM pol β in the presence of 250 nM Mus81/Eme1 and 10 nM FEN1. Lanes 10 and 20 correspond to synthesized size markers (M). Substrates were ^32^P-labeled at the 5′-end of their upstream strands. (**B**) Mus81/Eme1 cleavage of the upstream flap of a CAG repeat double-flap intermediate was examined using a double-flap substrate with an upstream 3′-(CAG)_4_ and a downstream 5′-(CAG)_3_-THF flap (left panel) or a substrate with an upstream 3′-(CAG)_7_ flap and a downstream 5′-(CAG)_7_-THF flap (right panel). The substrates were radiolabeled at the 3′-end of the upstream strand. Lanes 1 and 7 represent a substrate alone. Lanes 2 and 8 represent reaction mixtures with 250 nM Mus81/Eme1. Lanes 3 and 9 correspond to reaction mixtures with 250 nM Mus81/Eme1 and 5 nM pol β. Lanes 4 and 10 represent reaction mixtures with 250 nM Mus81/Eme1 and 10 nM FEN1. Lanes 5 and 11 correspond to reaction mixtures with 250 nM Mus81/Eme1, 5 nM pol β and 10 nM FEN1. Lanes 6 and 12 represent synthesized size markers (M). The substrates are illustrated schematically above the gels. A scheme that indicates Mus81/Eme1 cleavage of the upstream 3′-flap of the double-flap substrates is illustrated below the gels.

Because removal of an 8-oxoG and 5′-incision of an abasic lesion in a hairpin loop may further convert the hairpin into an ssDNA break intermediate with an upstream 3′-flap and a downstream 5′-flap, cleavage of the flaps by a 3′-5′ flap endonuclease such as Mus81/Eme1 and 5′-flap endonuclease FEN1 may remove the hairpin and produce the unexpanded product. To test this possibility, we initially characterized the activity of the 3′-5′ flap endonuclease Mus81/Eme1 (31,33–37) in the context of a hairpin loop during BER. We found that Mus81/Eme1 endonucleolytically cleaved the (CAG)_7_ (Figure 1, lanes 9–12; Figure 2, lanes 7–10) and (CAG)_14_ hairpin substrates (Figure 1, lanes 22–25; Figure 2, lanes 18–21), resulting in a series of 3′-flap cleavage products. These products were not from the cleavage of the entire hairpins because no Mus81/Eme1 cleavage products were detected from a (CAG)_7_ or (CAG)_14_ hairpin substrate in the absence of OGG1 and APE1 (Supplementary Figure S4, lanes 2,4,6 and 8). This indicates that BER in the (CAG)_7_ and (CAG)_14_ hairpin loop regions converted the hairpins into intermediates with a 3′-flap that was subsequently cleaved by Mus81/Eme1. Because the Mus81/Eme1 cleavage products from (CAG)_7_ and (CAG)_14_ hairpin substrates correspond to fragments containing a part of the 20-nt random sequence flanking the (CAG)_7_ (Figure 1, lane 9; Figure 2, lane 7) and (CAG)_14_ hairpin (Figure 1, lane 22; Figure 2, lane 18); this suggests that a downstream 5′-(CAG)_4_ or (CAG)_7_ repeat flap annealed to the template and displaced the upstream strand, creating a long flap that was subsequently removed by Mus81/Eme1. This further resulted in removal of the hairpins and production of the unexpanded product. To further confirm this, we determined if Mus81/Eme1 cleavage on a double-flap substrate with an upstream 3′-(CAG)_4 _- and a downstream 5′-(CAG)_3_-flap or a substrate with a 3′-(CAG)_7_-flap and 5′-(CAG)_7_-flap, could also result in the unexpanded product. The substrates mimic double-flap intermediates converted from APE1-incised hairpins with varying sizes. To verify the presence of the 3′- and 5′-flaps in the substrates, we used Mung Bean Nuclease to probe the formation of 3′- and 5′-flaps of the double-flap substrates. The results showed that the nuclease cleavage of the upstream strand of the (CAG)_3_/(CAG)_4_ double-flap substrate generated products with 29–38 nt indicative of cleavage of 1–4 CAG repeats (Supplementary Figure S5, lanes 2–4). The nuclease cleavage of the downstream strand of the substrate resulted in products with 30–36 nt indicative of cleavage of 1–3 CAG repeats (Supplementary Figure S5, lanes 7–9). This indicates that the substrate contained an upstream (CAG)_4_ flap and a downstream 5′-(CAG)_3_-THF flap (Supplementary Figure S5, the flap substrates illustrated at the bottom). Mung Bean Nuclease cleavage on the upstream strand of the (CAG)_7_ double-flap substrate resulted in products with 29–46 nt (Supplementary Figure S6, lanes 2–5). The nuclease cleavage on the downstream strand of the substrate generated products with 30–47 nt (Supplementary Figure S6, lanes 8–11). The results indicate that the nuclease cleaved 1–7 CAG repeats in both the upstream and downstream strands demonstrating the formation of a (CAG)_7_-flap in both of the strands (Supplementary Figure S6, the flap substrates illustrated at the bottom). We found that the Mus81/Eme1 cleavage pattern on the double-flap substrates was similar to that on the (CAG)_7_ and (CAG)_14_ hairpin substrates (Figure 3A, lanes 6–7 and lanes 16–17), indicating that the hairpins were indeed converted to the double-flap intermediates that were processed by Mus81/Eme1 during BER. Interestingly, we found that for the large (CAG)_14_ hairpin and its corresponding double-flap intermediate, Mus81/Eme1 cleavage in the presence of FEN1 mainly resulted in relatively larger products compared with its cleavage in the absence of FEN1 (Figure 1, compare lane 24 with lane 22; Figure 2, compare lane 20 with lane 18; Figure 3A, compare lane 18 with lane 16). This indicates that Mus81/Eme1 cleaved a shorter 3′-flap in the presence of FEN1 than it did in the absence of FEN1. This suggests that removal of a 5′-flap by FEN1 allowed the upstream 3′-flap to anneal to the template, resulting in a short 3′-flap that was cleaved by Mus81/Eme1. To further determine the number of Mus81/Eme1 cleavage products, the double-flap substrates were radiolabeled at the 3′-end of the upstream strand and incubated with Mus81/Eme1 in the absence and presence of FEN1 or/and pol β (Figure 3B). The results showed that in the absence of FEN1, Mus81/Eme1 flap cleavage on the (CAG)_3_/(CAG)_4_ double-flap substrate resulted in two products with 21 and 22 nt, respectively (Figure 3B, lanes 2–3), indicating that the 3′-flap endonuclease cleaved within the random sequence region that flanked the repeated sequence and removed the entirety of the 3′-repeat-containing flap. In the presence of FEN1, Mus81/Eme1 cleavage resulted in a 1-nt product (Figure 3B, lanes 4–5). This further confirmed that FEN1 removed a 5′-(CAG)_3_-THF flap before Mus81/Eme1 removal of a 3′-(CAG)_4_-flap allowing the 3′-flap to anneal to the template to create a 1-nt 3′-flap that was subsequently removed by Mus81/Eme1. For the (CAG)_7_ double-flap substrate, Mus81/Eme1 cleavage resulted in multiple products with 9–31 nt (Figure 3B, lanes 8–11), indicating that the 3′-(CAG)_7_ flap folded into a series of intermediates with a small hairpin attached to a short flap that was captured and cleaved by Mus81/Eme1. In the presence of FEN1, Mus81/Eme1 cleavage mainly resulted in products with 9–10 nt (Figure 3B, lanes 10–11). Again, this confirmed that FEN1 removed the downstream 5′-CAG repeat flap allowing the upstream flap to anneal to the template strand creating a short flap with 9 or 10 nt that was then cleaved by the 3′-endonuclease. In conclusion, our results demonstrate that BER in a CAG repeat hairpin loop can convert the hairpin into a double-flap intermediate with a 3′-flap and 5′-flap, which can be cleaved by a 3′-5′ and 5′-3′ flap endonuclease, such as Mus81/Eme1 and FEN1, in a cooperative manner resulting in removal of the hairpin and prevention of repeat expansion.

### FEN1 processed a CAG repeat hairpin by removing a 5′-CAG repeat flap resulting in attenuation of repeat expansion during BER

**Figure 4.**
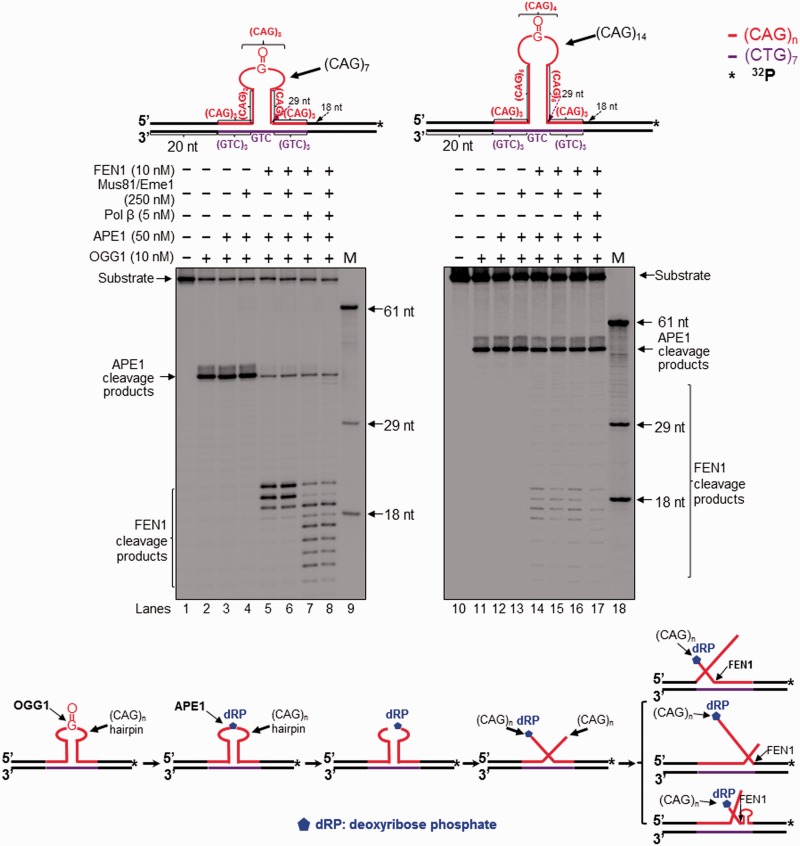
FEN1 processing of a hairpin during repair of an 8-oxoG in the hairpin loop. FEN1 flap cleavage on a CAG hairpin with an 8-oxoG in the loop region was examined with substrates containing a (CAG)_7_ (left panel) or (CAG)_14_ hairpin (right panel) with an 8-oxoG in the hairpin loop. Lanes 1 and 10 indicate substrates only. Lanes 2 and 11 correspond to reaction mixtures with 10 nM OGG1. Lanes 3 and 12 represent reaction mixtures with 10 nM OGG1 and 50 nM APE1. Lanes 4 and 13 represent reaction mixtures with 10 nM OGG1, 50 nM APE1 and 250 nM Mus81/Eme1. Lanes 5–6 and 14–15 correspond to reaction mixtures with and without 250 nM Mus81/Eme1 in the presence of 10 nM OGG1, 50 nM APE1 and 10 nM FEN1. Lanes 7–8 and 16–17 represent reaction mixtures with or without 250 nM Mus81/Eme1 in the presence of 10 nM OGG1, 50 nM APE1, 10 nM FEN1 and 5 nM pol β. Lanes 9 and 18 correspond to a series of synthesized size markers (M) for illustrating the size of FEN1 cleavage products. Substrates were ^32^P-labeled at the 3′-end of the hairpin-containing strand and are illustrated schematically above the gels. A scheme that indicates FEN1 cleavage activity in processing a CAG repeat hairpin with an 8-oxoG is illustrated below the gels.

**Figure 5.**
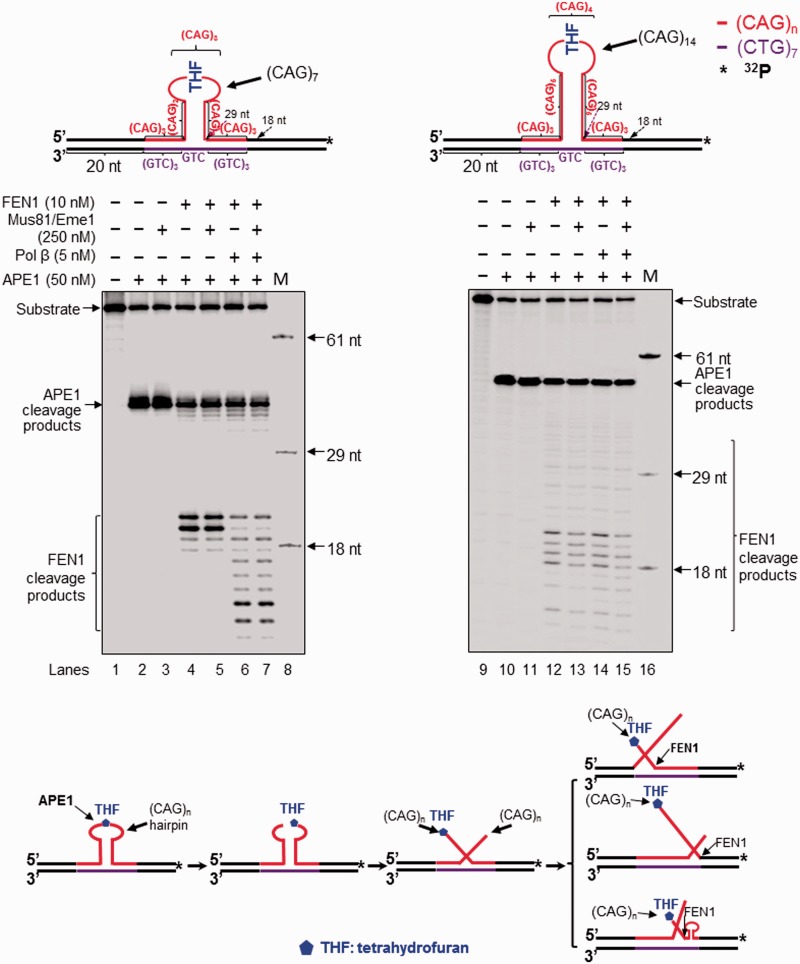
FEN1 processing of a hairpin during repair of an abasic site in the hairpin loop. FEN1 flap cleavage on a CAG hairpin containing an abasic site in the loop region was examined with substrates containing a (CAG)_7_ (left panel) or (CAG)_14_ hairpin (right panel) with a THF in the hairpin loop. Lanes 1 and 9 represent substrates only. Lanes 2 and 10 correspond to reaction mixtures with 50 nM APE1. Lanes 3 and 11 correspond to reaction mixtures with 50 nM APE1 and 250 nM Mus81/Eme1. Lanes 4–5 and 12–13 correspond to reaction mixtures with 50 nM APE1 and 10 nM FEN1 in the absence or presence of 250 nM Mus81/Eme1. Lanes 6–7 and 14–15 correspond to reaction mixtures with or without 250 nM Mus81/Eme1 in the presence of 50 nM APE1, 10 nM FEN1 and 5 nM pol β. Lanes 8 and 16 correspond to a series of synthesized size markers (M). Substrates were ^32^P-labeled at the 3′-end of the hairpin containing strands and are illustrated schematically above the gels. A scheme that indicates FEN1 cleavage activity in processing a CAG repeat hairpin with a THF residue is illustrated below the gels.

**Figure 6.**
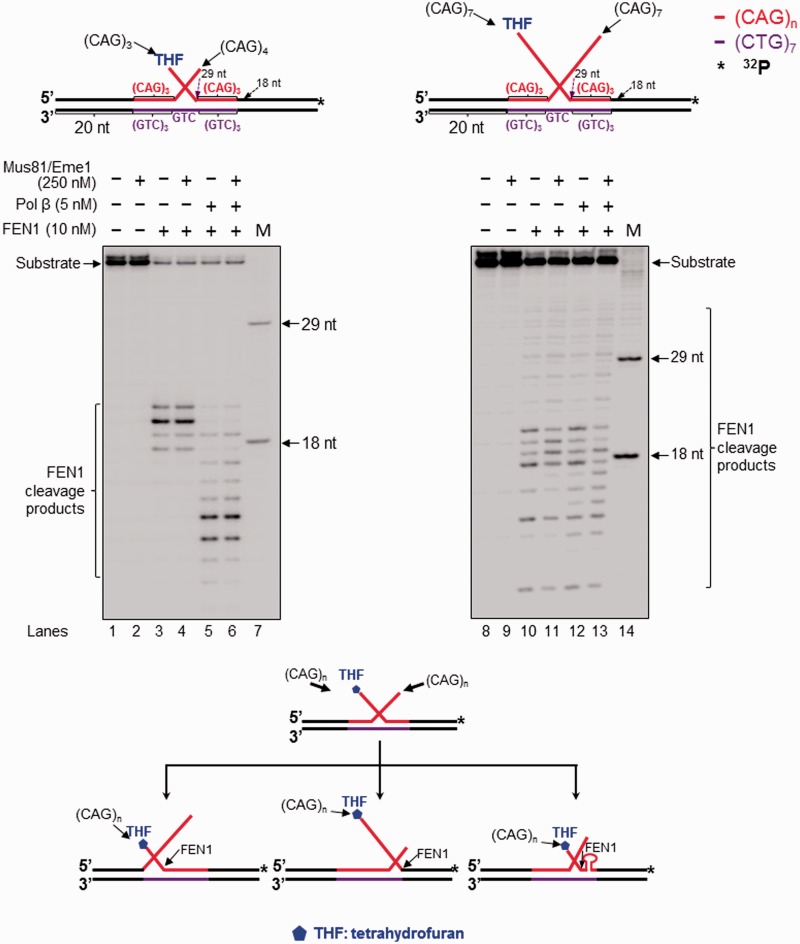
FEN1 cleavage of CAG repeat double-flap intermediates. FEN1 cleavage of CAG repeat double-flap intermediates resulting from APE1 incision at a CAG hairpin loop region was examined with a substrate containing a 3′-(CAG)_4_ and a 5′-THF-(CAG)_3_ (left panel) and a substrate with a 3′-(CAG)_7_ flap and a 5′-THF-(CAG)_7_ flap (right panel). Lanes 1 and 8 correspond to substrates only. Lanes 2 and 9 correspond to reaction mixtures with 250 nM Mus81/Eme1. Lanes 3–4 and 10–11 correspond to reaction mixtures with or without 250 nM Mus81/Eme1 in the presence of 10 nM FEN1. Lanes 5–6 and 12–13 correspond to reaction mixtures with and without 250 nM Mus81/Eme1 in the presence of 10 nM FEN1 and 5 nM pol β. Lanes 7 and 14 correspond to synthesized size markers (M). Substrates were ^32^P-labeled at the 3′-end of their downstream strands. A scheme that indicates FEN1 cleavage activity in removing a double-flap with a 5′-THF is illustrated below the gels.

FEN1 plays a dual role in modulating TNR stability by processing a 5′-TNR flap/hairpin (17,20,38,39). Coordination between FEN1 and pol β leads to both TNR expansion and deletion during BER (20,21). It is possible that FEN1 may facilitate removal of a hairpin by processing a 5′-CAG repeat flap during BER in a hairpin loop, preventing CAG repeat expansion. To test this possibility, we examined FEN1 cleavage during BER in a CAG repeat hairpin loop and a double-flap intermediate. We found that FEN1 cleavage on the (CAG)_7_ hairpin substrate resulted in the unexpanded product (Figure 1, lanes 5 and 7; Figure 2, lanes 3 and 5), indicating that the enzyme removed the entire hairpin. Interestingly, FEN1 cleavage on the (CAG)_14_ hairpin resulted in both the unexpanded product and expanded products that were shorter than the expanded/uncut substrate strand (Figure 1, lanes 18–21; Figure 2, lanes 14–17). This indicates that FEN1 cleavage completely removed a small and large CAG repeat hairpin, but also removed a part of a large hairpin during BER. To further determine how FEN1 can remove a hairpin by flap cleavage on the hairpin substrates, we examined FEN1 cleavage using the substrates that were radiolabeled at the 3′-end of the hairpin-containing expanded/uncut strand. The results showed that in the absence of pol β, FEN1 efficiently cleaved a 5′-(CAG)_6_ flap from the (CAG)_7_ hairpin substrate (Figure 4, lane 5; Figure 5, lane 4), but removed a 5′-(CAG)_10_ flap and other shorter 5′-flaps from (CAG)_14_ hairpin substrates with a low efficiency (Figure 4, lane 14; Figure 5, lane 12). This suggests that BER in the loop of a small hairpin generated a short 5′-flap that was efficiently cleaved by FEN1 resulting in removal of the entirety of a small hairpin. On the other hand, BER in a large hairpin generated a long 5′-flap that inhibited FEN1 cleavage. This forced FEN1 to use its alternate flap cleavage activity to capture and cleave a short 5′-flap, thereby resulting in partial removal of a large hairpin. This was further supported by the fact that FEN1 alternate cleavage resulted in multiple products (The right panel of Figures 4–6), indicating that the enzyme captured and cleaved a series of short flaps. This suggests that a long (CAG)_7_ flap folded into multiple small alternate hairpins attached to a short flap of varying sizes. FEN1 then loaded from the 5′-end of the short flap and tracked down to the bottom of the flaps and cleaved them. To determine if all FEN1 products resulted from its flap cleavage activity, we examined its cleavage on a (CAG)_7_ or (CAG)_14_ hairpin substrate in the absence of OGG1 and APE1. We failed to observe any FEN1 cleavage products from the substrates (Supplementary Figure S7, lanes 2, 4, 6, and 8), indicating that FEN1 cannot directly cleave a hairpin, and that the products were from the flap cleavage activity of the enzyme. This further demonstrates a critical role of FEN1 flap cleavage in removing hairpin structures during BER. In the presence of pol β, FEN1 cleavage on the small (CAG)_7_ hairpin substrate was stimulated (Figure 4, compare lane 5 with lane 7; Figure 5, compare lane 4 with lane 6), presumably by pol β strand-displacement synthesis that generated a 5′-flap. However, FEN1 cleavage on the (CAG)_14_ hairpin substrate was not affected by pol β synthesis (Figure 4, compare lane 14 with lane 16; Figure 5, compare lane 12 with lane 14), suggesting that a long 3′-flap was formed during BER, and this inhibited pol β DNA synthesis. Consistent with this, FEN1 cleavage on the double-flap substrates that mimic flap intermediates from an incised hairpin loop resulted in the same products as those from its cleavage on the hairpin substrates (Figure 6, lanes 3–6 and 10–13). This confirmed that BER in a hairpin loop generated a 5′-CAG-flap that was cleaved by FEN1. Because Mus81/Eme1 can stimulate FEN1 flap cleavage (40), we then asked if Mus81/Eme1 can stimulate FEN1 cleavage on the (CAG)_7_ and (CAG)_14_ hairpin substrates. We found that Mus81/Eme1 failed to stimulate FEN1 cleavage on the substrates (Figure 4, compare lane 7 with lane 8, lane 16 with lane 17; Figure 5, compare lane 6 with lane 7, lane 14 with lane 15; Figure 6, compare lane 5 with lane 6, lane 12 with lane 13). This suggests that Mus81/Eme1 cleavage on a 3′-flap does not affect FEN1 processing of a CAG repeat hairpin.

### Pol β gap-filling synthesis facilitated the production of the unexpanded product during BER of a base lesion on a large CAG hairpin loop

As a central component of BER, pol β fills in gaps, performs strand-displacement synthesis and modulates TNR stability during BER (32). Thus it may also play a critical role during BER in a hairpin loop. We found that pol β stimulated the production of the unexpanded product from the large (CAG)_14_ hairpin (Figure 1, lanes 20 and 21; Figure 2, lanes 16 and 17), but not that from the small (CAG)_7_ hairpin (Figure 1, lanes 7 and 8; Figure 2, lanes 5 and 6). The absence of pol β reduced the production of the unexpanded product from the large hairpin (Figure 1, lanes 18 and 19; Figure 2, lanes 14 and 15) and the long double-flap substrates (Figure 3A, lanes 12 and 13), indicating that pol β is critical for removing the entirety of a large hairpin. In addition, pol β performed more efficient DNA synthesis during BER in the small hairpin loop (Figure 1, lanes 10 and 12; Figure 2, lanes 8 and 10) and the short double-flap (Figure 3A, lanes 7 and 9) than in the large hairpin loop (Figure 1, lanes 23 and 25; Figure 2, lanes 19 and 21) and the long double-flap (Figure 3A, lanes 17 and 19). This indicates that a short 3′-flap from APE1 5′-incision of a small hairpin readily annealed to its template strand and was efficiently extended by pol β, whereas a long 5′-flap from a large hairpin partially annealed to its template by folding into an intermediate with a small hairpin, thus inhibiting pol β DNA synthesis. Our results further demonstrate that pol β was capable of performing DNA synthesis with the (CAG)_3_/(CAG)_4_ double-flap substrate in the absence of Mus81/Eme1 (Figure 3A, lane 4). This indicates that a small (CAG)_7_ hairpin was readily converted into a short flap during BER and was subsequently fully annealed to the template strand leaving a base-paired 3′-terminus for pol β DNA synthesis without need of Mus81/Eme1 cleavage. However, for a large (CAG)_14_ hairpin, pol β failed to synthesize DNA in the absence of Mus81/Eme1 (Figure 3A, lane 14), indicating that the long flap formed from a large hairpin failed to completely anneal to the template strand, leaving a short 3′-flap that prevented pol β DNA synthesis and needs to be removed by Mus81/Eme1. This further demonstrates that removal of a 3′- or 5′-flap by Mus81/Eme1 and FEN1 plays a crucial role in facilitating pol β DNA synthesis (Figure 3A, lane 9 and lane 19). Thus, we suggest that pol β DNA synthesis can be hindered by a 3′-flap rather than by the Mus81/Eme1 protein complex during BER in hairpin loops. Removal of a 3′-flap by Mus81/Eme1 is essential in facilitating pol β DNA synthesis during BER in a large hairpin.

## DISCUSSION

**Figure 7.**
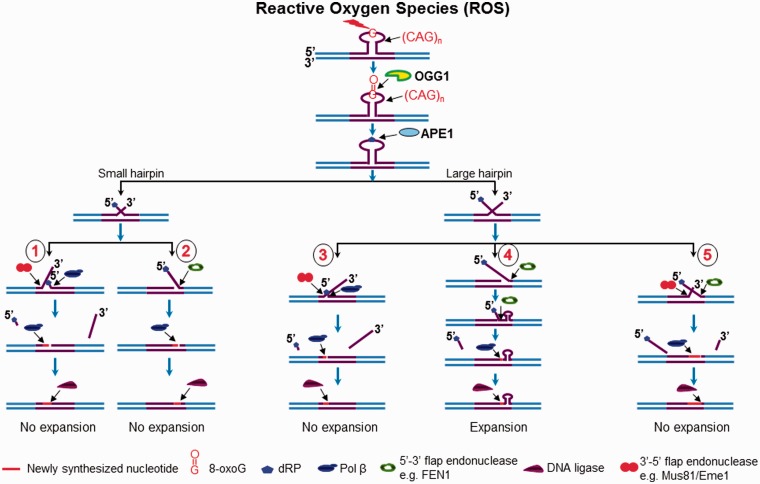
Prevention or attenuation of CAG repeat expansion by BER coupled with removal of a hairpin. Oxidative stress can induce an oxidized DNA base lesion, 8-oxoG in the loop region of a CAG hairpin. OGG1 removes 8-oxoG leaving an abasic site that is subsequently 5′-incised by APE1 generating ssDNA break in the hairpin loop. This further results in the formation of a double-flap intermediate with a 3′- and 5′-CAG repeat flap. For a small hairpin, APE1 5′-incision leads to the formation of a double-flap intermediate with a short 3′- and 5′- flap that can be completely cleaved by a 3′-5′ endonuclease, such as Mus81/Eme1, or by FEN1 flap cleavage. The flap cleavage can result in a gap that is filled by pol β DNA synthesis and this completes damage repair. This results in prevention of repeat expansion (sub-pathway 1 and 2). Repair of a base lesion located in the loop region of a large CAG repeat hairpin results in the formation of a relatively long 3′- and 5′-flap. The 5′-flap can anneal to the template strand to create a long 3′-flap that is cleaved by a 3′-5′ endonuclease such as Mus81/Eme1, thereby preventing repeat expansion (sub-pathway 3). A 3′-flap can also anneal to its template to create a long 5′-flap for FEN1 cleavage. FEN1 cleaves the 5′-flap either by its conventional flap cleavage to remove the entire hairpin or by its alternate flap cleavage to partially remove the hairpin. This results in prevention or attenuation of repeat expansion (sub-pathway 3 and 4). A double-flap intermediate can also be processed simultaneously by Mus81/Eme1 and FEN1 leading to the prevention of repeat expansion (sub-pathway 5).

In this study, we provide the first evidence that 8-oxoG in the loop region of a CAG repeat hairpin in a CAG repeat tract can be removed by OGG1, leaving an abasic site that can be subsequently incised by APE1 (Figures 1 and 2). We demonstrate that OGG1 removes a base lesion located in the loop region of a small (CAG)_7_ hairpin more efficiently than that in a large (CAG)_14_ hairpin loop (Figure 1, compare lanes 2–3 with lanes 15–16). Furthermore, we found that 5′-incision of an abasic site in the loop region of a hairpin resulted in an ssDNA break in the loop that was repaired by BER, leading to partial or complete removal of the hairpin (Figures 1 and 2). This indicates that BER in the loop of a hairpin can be coupled with the removal of the hairpin. We show that BER in a CAG hairpin loop results in removal of the hairpin, leading to attenuation of CAG repeat expansion. This is accomplished by the coordination among FEN1 cleavage of a 5′-flap, cleavage of a 3′-flap by a 3′-5′ endonuclease such as Mus81/Eme1 and pol β DNA synthesis (Figures 1–3). We further demonstrate that as a 3′-5′ flap endonuclease, Mus81/Eme1 removed both a small (CAG)_7_ hairpin and a large (CAG)_14_ hairpin by cleaving a 3′-CAG repeat flap (Figures 1–3). FEN1 5′-flap cleavage resulted in complete removal of both a small and large hairpin, as well as partial removal of a large hairpin (Figures 1–3). This was demonstrated by the fact that FEN1 cleavage resulted in the unexpanded product (Figures 1–3) as well as a series of shortened expanded products (The right panel of Figures 1–3). These results indicate that a single-strand break in the loop region of a CAG repeat hairpin converts the hairpin into an intermediate with a 3′- and 5′-flap that can be cleaved by a 3′-5′ endonuclease or FEN1. This further suggests that a long 5′-flap can fold into a small hairpin with a short 5′-flap that is cleaved by FEN1 alternate flap cleavage activity. Finally, we found that the presence of both Mus81/Eme1 and FEN1, along with pol β, significantly stimulated the production of the unexpanded product, indicating that a 3′-5′ endonuclease such as Mus81/Eme1 can cooperate with FEN1 and pol β to remove a hairpin during BER, thereby preventing CAG repeat expansion. Our results support a model that is shown in Figure 7, in which an 8-oxoG in the loop region of a CAG repeat hairpin is induced by oxidative DNA damage. OGG1 removes the base lesion, leaving an abasic site that can be 5′-incised by APE1. This results in a single-strand break in the loop region of the hairpin, converting the cleaved hairpin into a double-flap intermediate with a 3′-flap and a 5′-flap. For a small hairpin, a short 3′-flap and a 5′-flap are generated and cleaved by the 3′-5′ endonuclease Mus81/Eme1 and the 5′-3′ endonuclease FEN1. This leads to a complete removal of the hairpin via BER of an 8-oxoG in the hairpin loop, thereby preventing repeat expansion (Figure 7, sub-pathways 1 and 2). Repair of an 8-oxoG in a large hairpin loop results in a double-flap intermediate with a long 3′-flap and 5′-flap. The 3′-5′ endonuclease Mus81/Eme1 removes the entirety of the hairpin by cleaving the 3′-flap, preventing repeat expansion (Figure 7, sub-pathway 3). FEN1 processes the 5′-flap either by its conventional flap cleavage to completely remove the hairpin or by its alternate cleavage activities to partially remove the hairpin. This results in prevention or attenuation of repeat expansion (Figure 7, sub-pathway 4). Simultaneous cleavage of a 3′-flap and a 5′-flap by Mus81/Eme1 and FEN1, in cooperation with pol β gap-filling synthesis, prevents FEN1 alternate flap cleavage, thereby promoting the prevention of repeat expansion (Figure 7, sub-pathway 5).

Removal of hairpin structures has been shown to be a challenge to various biological systems, in particular to eukaryotes. In *E. coli*, a structure-specific endo/exonuclease complex SbcCD has been found to endonucleolytically incise the loop region of a CAG/CTG hairpin by using its endonuclease activity, and then exonucleolytically cleave the hairpin stem with its dsDNA exonuclease activity (41,42). In bacteriophage T7, endonuclease I can cleave a looped-out CAG/CTG repeat bubble structure, raising the possibility that this enzyme may also cleave a CAG/CTG repeat hairpin (43). However, in yeast, CTG and CAG repeat hairpin structures can readily escape cellular repair mechanisms, leading to repeat expansion (44). In human cell extracts, three different paths for resolving a hairpin have been proposed, depending on the location of the hairpin. A CTG hairpin or bubble (named slipped repeats or slip-out) formed in a nascent DNA strand with a 5′-nick can be partially removed, leading to repeat expansion (45,46). A hairpin opposite a 5′-nick in the template strand results in complete removal of the hairpin and maintenance of repeat length (45,46). This may be mediated by destabilization of a template hairpin by DNA polymerase passing-through of the hairpin via DNA synthesis (20) or by a DNA helicase (47). A hairpin opposite a 3′-nick in the template strand can lead to repeat deletion (45,46). It has been found that the mismatch repair protein complexes, MSH2/MSH3 (MutSβ), MutLα and PMS2 may mediate the removal of small (CAG)_1_ -(CAG)_3_ hairpins/slip-outs (48), but not for large hairpins (49,50), suggesting an important role of mismatch repair in removing small TNR hairpins during DNA replication and repair. In this study, for the first time, we identified BER as a new pathway for removing CAG repeat hairpins in a CAG repeat tract via repair of a DNA base lesion in the hairpin loop region. Our results indicate that BER in CAG hairpin loops incises the hairpin loop region, converting the hairpins into intermediates with a 5′- and a 3′-flap that are cleaved by FEN1 and the 3′-5′ flap endonuclease, Mus81/Eme1. This ultimately results in removal of a CAG repeat hairpin and prevention or attenuation of TNR expansion. Thus, here we suggest a unique pathway for removing TNR hairpins, which is induced by a base lesion and mediated by BER at the hairpin loop region.

Our study demonstrates an important role of the flap cleavage activity of both 3′-5′ and 5′-3′ endonucleases such as Mus81/Eme1 and FEN1 in preventing or attenuating TNR expansion by removing hairpin structures through coupling with BER. Our discovery is supported by a finding that the nucleotide excision repair protein XPG, which is also a 5′-3′ flap endonuclease (51), can promote removal of a hairpin through a 5′-incision (52). It is also supported by the fact that the 3′-5′ endonuclease/exonuclease Mre11-Rad50-Nbs1 complex can facilitate the removal of CAG/CTG hairpins *in vitro* and *in vivo* (53). The 3′-5′ exonuclease activity of Mre11 was initially identified as an enzymatic activity that resects the 3′-end of a double-stranded DNA for repairing double-strand DNA breaks (54,55). It is possible that this activity of Mre11 may also cleave TNRs from the 3′-end of an upstream TNR-containing strand, removing an extra number of the repeats. This further allows the downstream repeat flap to anneal to its template, thereby promoting removal of a hairpin and prevention of repeat expansion. It is conceivable that other 3′-5′ and 5′-3′ endo/exonucleases may also use the same mechanisms as the ones exemplified by Mus81/Eme1 and FEN1 to prevent TNR expansion.

Our results showed that more APE1 incision products than OGG1 products were produced (Figure 1, compare lanes 2 and 15 with lanes 3 and 16), indicating that OGG1 removed an 8-oxoG in a hairpin loop more efficiently in the presence of APE1 than in the absence of the enzyme. This is consistent with the notion that APE1 can facilitate OGG1 incision of an 8-oxoG by kicking OGG1 off an abasic site, releasing OGG1 from its product, and thereby increasing OGG1 recycling and its efficiency for removing oxidative DNA damage (56,57). This indicates that APE1 can also stimulate OGG1 incision of 8-oxoG by dislodging the enzyme from an abasic site located in a hairpin loop. Thus, the coordination between OGG1 and APE1 in removing DNA base lesions is conserved in both duplex DNA and hairpins. BER cofactors that can stimulate OGG1 and APE1 activity may stimulate removal of hairpins during BER, thereby facilitating prevention or attenuation of repeat expansion. For example, BER cofactor X-ray repair cross-complementing protein 1 (XRCC1) physically interacts with OGG1 and stimulates its activity by 2 - to 3-fold, presumably by recruiting OGG1 to an 8-oxoG (58,59). High mobility group box 1B (HMGB1) can stimulate APE1 5′-incision of an abasic site (60). Thus these cofactors may promote removal of hairpins and prevention of repeat expansions by stimulating OGG1 and APE1 activity. The coordination among OGG1, XRCC1, APE1 and HMGB1 in repairing a base lesion in the context of a TNR hairpin that is coupled with removal of a hairpin, remains to be elucidated.

In this study, we showed that BER enzymes, pol β and FEN1 can coordinate with a 3′-5′ flap endonuclease represented by Mus81/Eme1 to remove a relatively large CAG repeat hairpin during BER in the hairpin loop region. The repair process is accomplished by the coordination among Mus81/Eme1 cleavage on a 3′-flap, FEN1 removal of a 5′-flap and pol β gap-filling synthesis. We found that FEN1 alone resulted in the unexpanded product as well as a series of shortened expansion products, indicating that FEN1 can cleave a part of a long CAG flap using its alternate flap cleavage activity. This activity of FEN1 can be inhibited by its cooperation with a 3′-5′ flap endonuclease such as Mus81/Eme1, thereby promoting the complete removal of the CAG repeat hairpin and prevention of repeat expansion. Thus, the cooperation between FEN1 and a 3′-5′ flap endonuclease plays a critical role in preventing CAG repeat expansion during BER. Because the production of the unexpanded product was also stimulated by pol β (Figure 1, compare lane 19 with lane 21; Figure 2, compare lane 15 with lane 17; Figure 3A, compare lane 13 with lane 15), this further indicates that pol β fills in a gap resulting from the 3′- and 5′-flap cleavage by Mus81/Eme1 and FEN1, creating a ligatable nick for DNA ligase to complete the repair. Thus, it appears that the coordination among FEN1, the 3′-5′ flap endonuclease Mus81/Eme1 and pol β plays a crucial role in removing a large CAG repeat hairpin and prevention of CAG repeat expansion.

Interestingly, we found that for a small hairpin with a base lesion in the loop region, FEN1 alone can completely remove the downstream flap to produce the non-expansion product, thereby preventing repeat expansion (Figure 1, lane 5; Figure 2, lane 3; Figure 3A, lane 2). This is because a short upstream flap can anneal back to the template and displace the downstream strand, creating a downstream 5′-flap. This is consistent with the fact that FEN1 can efficiently remove a small hairpin during BER by removing a short flap via flap equilibration, as demonstrated previously (61). For a large hairpin that can be converted to an intermediate with long double-flaps, FEN1 can use its conventional or alternate flap cleavage to completely or partially remove the hairpin. However, the efficiency of FEN1 in removing a large hairpin may be modulated by interacting with other replication and repair proteins. For example, the Bambara group has demonstrated that replication protein A (RPA) can inhibit FEN1 cleavage of a long flap with 30 nt but not a short flap (62,63). This may compromise the efficiency of FEN1 to remove a large hairpin. Yet, the inhibitory effect from RPA may be alleviated by the cooperation between FEN1 and Dna2, a replication helicase and nuclease (64). This is because binding of RPA to the long flap can stimulate Dna2 cleavage on the flap, which shortens it. This allows FEN1 to readily load onto the short flap and remove it completely (64). It is conceivable that during BER of a base lesion in a large hairpin, a long double-flap can be initially bound by RPA. This then stimulates Dna2 cleavage activity, which shortens a long 5′-flap. FEN1 subsequently cleaves the shortened 5′-flap efficiently, leading to complete removal of the large hairpin. Moreover, because BER cofactors and enzymes of other repair pathways such as proliferating cell nuclear antigen (PCNA) and the 5′-3′ exonuclease Exo I can facilitate FEN1 flap cleavage (65,66), it is possible that these proteins may also facilitate removal of a TNR hairpin. The cooperation among FEN1 and other BER proteins and cofactors, as well as the enzymes of other repair pathways in removing hairpin structures during BER needs to be elucidated.

In summary, in this study we discovered a unique pathway for removal of a TNR hairpin via BER in the loop region of the hairpin. We have demonstrated that OGG1 and APE1 can remove an 8-oxoG located at the hairpin loop region, leaving an abasic site that can be further incised by APE1, and resulting in incision of the hairpin and the conversion of the hairpin into an intermediate with a 3′-flap and a 5′-flap. For a small hairpin, FEN1 or a 3′-5′ endonuclease such as Mus81/Eme1 alone can completely remove the hairpin by cleaving a 5′-flap or a 3′-flap, whereas removal of a large hairpin via BER is mediated by the cooperation among the cleavage of a 5′-flap by FEN1, the cleavage of a 3′-flap by a 3′-5′ flap endonuclease and pol β gap-filling synthesis. Our study indicates that BER is coupled with the removal of a TNR hairpin to prevent or attenuate TNR expansion, and we suggest a new role of oxidative DNA damage and BER in attenuating TNR expansion by removing a CAG repeat hairpin.

## SUPPLEMENTARY DATA

Supplementary Data are available at NAR Online.

## FUNDING

National Institutes of Health (NIH) [ES017476 and ES023569 to Y.L.]; National Natural Science Foundation of China [81172632 to Z.Z.]; National Institutes of Health [HL105631 to Y.Z.]. Funding for open access charge: NIH.

*Conflict of interest statement*. None declared.

## Supplementary Material

Supplementary Data
