# Supplementary material for: Base excision repair of oxidative DNA damage coupled with removal of a CAG repeat hairpin attenuates trinucleotide repeat expansion
Source: Nucleic Acids Res. 2014 Jan 14;42(6):3675–91. doi: 10.1093/nar/gkt1372 (PMC3973345; doi:10.1093/nar/gkt1372)
Supplement: Supplementary Data [file supp_42_6_3675__index.html]

Base excision repair of oxidative DNA damage coupled with removal of a CAG repeat hairpin attenuates trinucleotide repeat expansion — Base excision repair of oxidative DNA damage coupled with removal of a CAG repeat hairpin attenuates trinucleotide repeat expansion — Supplementary Data 

# Base excision repair of oxidative DNA damage coupled with removal of a CAG repeat hairpin attenuates trinucleotide repeat expansion

## Supplementary Data

files

**Files in this Data Supplement:**

- Supplementary Data - pdf file
