# Supplementary material for: Base excision repair of oxidative DNA damage coupled with removal of a CAG repeat hairpin attenuates trinucleotide repeat expansion
Source: Nucleic Acids Res. 2014 Jan 14;42(6):3675–91. doi: 10.1093/nar/gkt1372 (PMC3973345; doi:10.1093/nar/gkt1372)
Supplement: Supplementary Data [file supp_gkt1372_nar-02123-f-2013-File010.pdf]

Table S1 Oligonucleotide sequence

Oligonucleotides	nt	Sequence (5’ –3’)
Downstream		
D1	40	p**F** CAG CAG CAG CAG CAG CAG TA CGT AGA CTT ACT CAT TGC
D2	50	p**F** CAG CAG CAG CAG CAG CAG CAG CAG CAG CAG TA CGT AGA CTT ACT CAT TGC
D3	79	CGA GTC ATC TAG CAT CCG TA CAG CAG CAG CAG CAG CAG CA **8oxo-G** CAG CAG CAG CAG CAG CAG TA CGT AGA CTT ACT CAT TGC
D4	100	CGA GTC ATC TAG CAT CCG TA CAG CAG CAG CAG CAG CAG CAG CAG CAG CA **8oxo-G** CAG CAG CAG CAG CAG CAG CAG CAG CAG CAG TA CGT AGA CTT ACT CAT TGC
D5	78	CGA GTC ATC TAG CAT CCG TA CAG CAG CAG CAG CAG CAG CA **F** CAG CAG CAG CAG CAG CAG TA CGT AGA CTT ACT CAT TGC
D6	100	CGA GTC ATC TAG CAT CCG TA CAG CAG CAG CAG CAG CAG CAG CAG CAG CA **F** CAG CAG CAG CAG CAG CAG CAG CAG CAG CAG TA CGT AGA CTT ACT CAT TGC
Template		
T1	61	GCA ATG AGT AAG TCT ACG TAC TGC TGC TGC TGC TGC TGC TGT ACG GAT GCT AGA TGA CTC G
Upstream		
U1	40	CGA GTC ATC TAG CAT CCG TA CA GCA GCA GCA GCA GCA GCA
U2	49	CGA GTC ATC TAG CAT CCG TA CAG CAG CAG CAG CAG CAG CAG CAG CAG CA

F: tetrahydrofuran, THF   8-oxoG: 8-oxoguanine

Fig. S1A

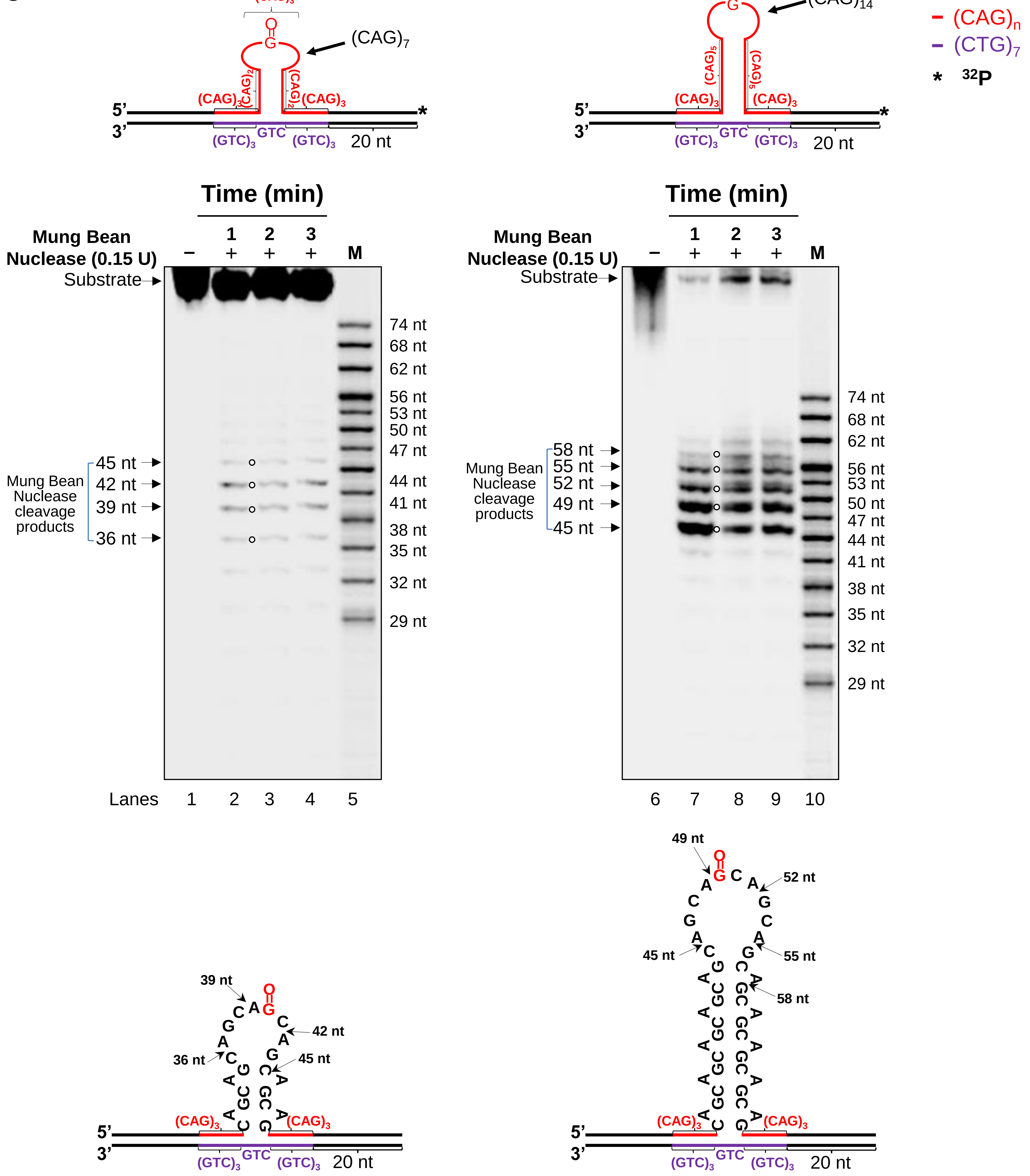
(CAG)3
(CAG)7
(CAG)2
(CAG)3
(CAG)3
5'
3'
(GTC)3
GTC
(GTC)3
20 nt
(CAG)4
(CAG)14
(CAG)5
(CAG)5
(CAG)3
(CAG)3
(GTC)3
GTC
(GTC)3
20 nt
(CAG)n
(CTG)7
* 32P
Time (min)
Mung Bean Nuclease (0.15 U)
1 2 3
– + + + M
Substrate
74 nt
68 nt
62 nt
56 nt
53 nt
50 nt
47 nt
44 nt
41 nt
38 nt
35 nt
32 nt
29 nt
Mung Bean Nuclease cleavage products
45 nt
42 nt
39 nt
36 nt
Lanes 1 2 3 4 5
Time (min)
Mung Bean Nuclease (0.15 U)
1 2 3
– + + + M
Substrate
58 nt
55 nt
52 nt
49 nt
45 nt
74 nt
68 nt
62 nt
56 nt
53 nt
50 nt
47 nt
44 nt
41 nt
38 nt
35 nt
32 nt
29 nt
6 7 8 9 10
39 nt
42 nt
36 nt
45 nt
49 nt
52 nt
45 nt
55 nt
58 nt

Fig. S1B

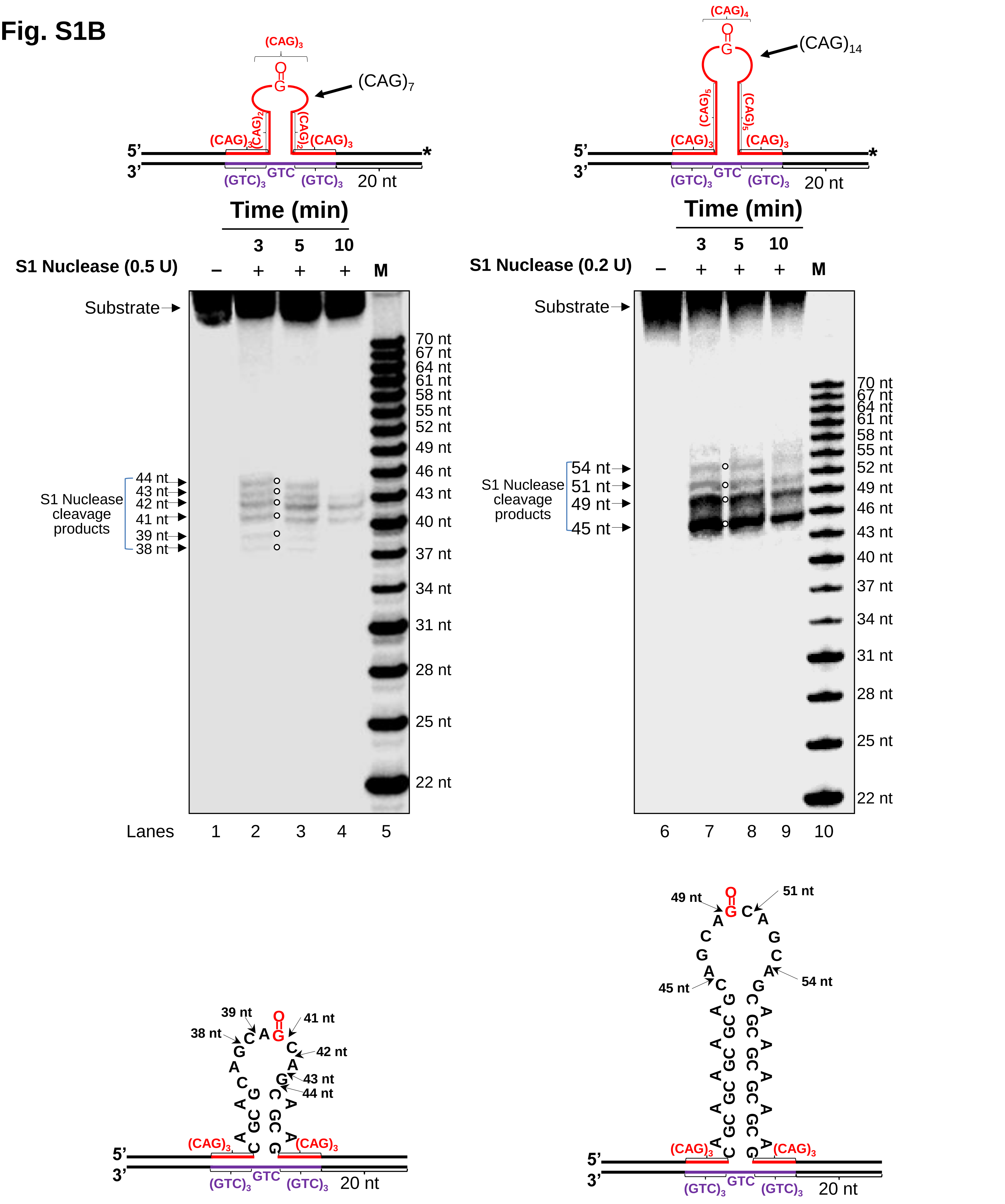

(CAG)7
(CAG)3
(CAG)14
(CAG)4
(CAG)5
5'
3'
(GTC)3
GTC
20 nt
Time (min)
3
5
10
S1 Nuclease (0.5 U)
S1 Nuclease (0.2 U)
M
Substrate
S1 Nuclease cleavage products
44 nt
43 nt
42 nt
41 nt
39 nt
38 nt
54 nt
51 nt
49 nt
45 nt
70 nt
67 nt
64 nt
61 nt
58 nt
55 nt
52 nt
46 nt
40 nt
37 nt
34 nt
31 nt
28 nt
25 nt
22 nt
Lanes
1
2
3
4
5
6
7
8
9
10

Fig. S2A

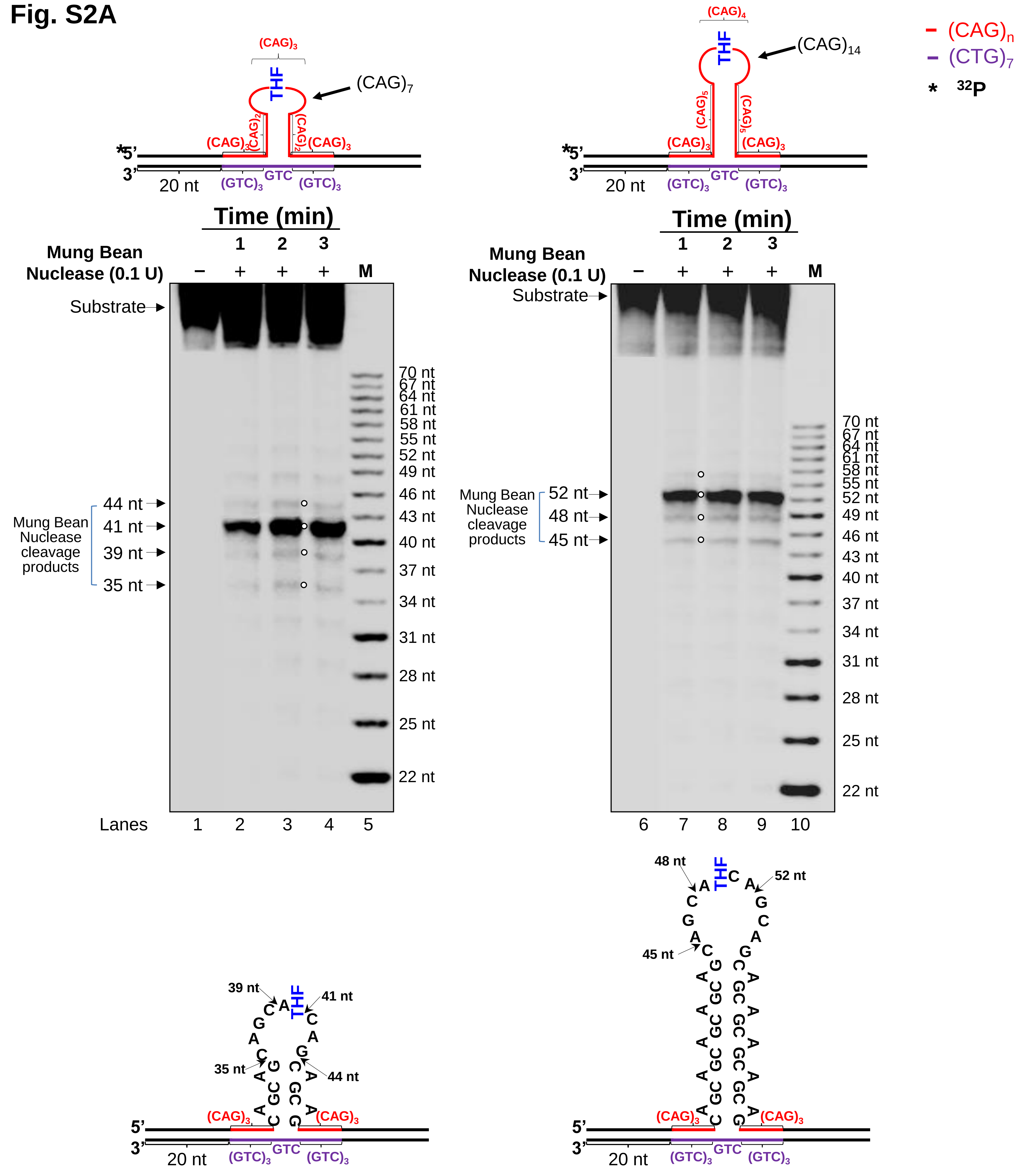

(CAG)3
THF
(CAG)7
(CAG)2
(CAG)3
*5'
3'
20 nt
(GTC)3
GTC
(GTC)3
(CAG)4
(CAG)14
(CAG)5
(CAG)n
(CTG)7
* 32P
Time (min)
1 2 3
Mung Bean Nuclease (0.1 U)
− + + + M
Substrate
Mung Bean Nuclease cleavage products
44 nt
41 nt
39 nt
35 nt
70 nt
67 nt
64 nt
61 nt
58 nt
55 nt
52 nt
49 nt
46 nt
43 nt
40 nt
37 nt
34 nt
31 nt
28 nt
25 nt
22 nt
52 nt
48 nt
45 nt
Lanes
1 2 3 4 5
6 7 8 9 10

Fig. S2B

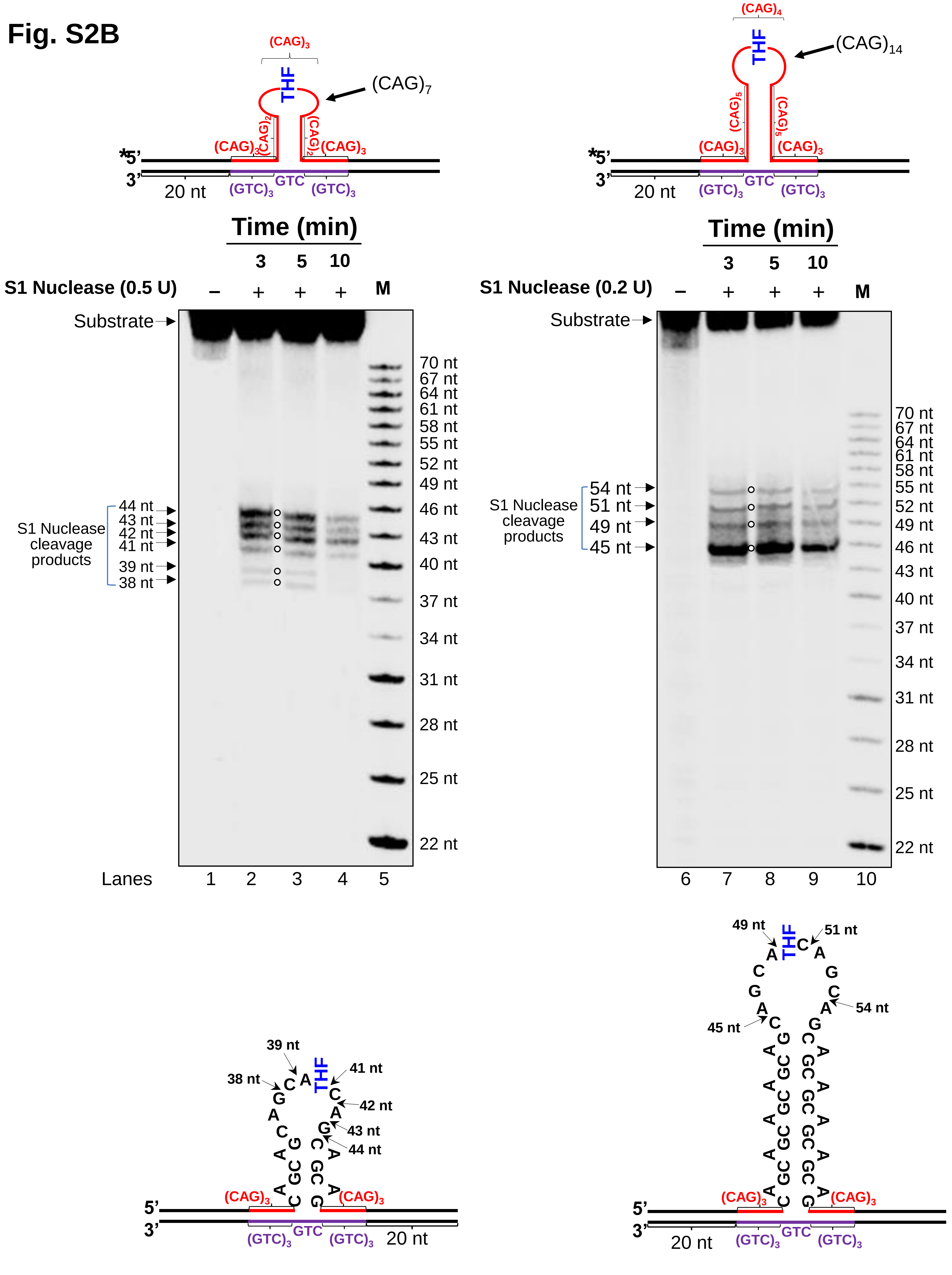

(CAG)3
THF
(CAG)7
(CAG)3
(CAG)2
(CAG)2
(CAG)3
*5'
3'
20 nt
(GTC)3
GTC
(GTC)3
Time (min)
3
5
10
S1 Nuclease (0.5 U)
–
+
+
+
M
Substrate
70 nt
67 nt
64 nt
61 nt
58 nt
55 nt
52 nt
49 nt
46 nt
43 nt
40 nt
37 nt
34 nt
31 nt
28 nt
25 nt
22 nt
S1 Nuclease cleavage products
44 nt
43 nt
42 nt
41 nt
39 nt
38 nt
Lanes
1
2
3
4
5
(CAG)4
THF
(CAG)14
(CAG)5
(CAG)5
(CAG)3
(CAG)3
*5'
3'
20 nt
(GTC)3
GTC
(GTC)3
Time (min)
3
5
10
S1 Nuclease (0.2 U)
–
+
+
+
M
Substrate
S1 Nuclease cleavage products
54 nt
51 nt
49 nt
45 nt
70 nt
67 nt
64 nt
61 nt
58 nt
55 nt
52 nt
49 nt
46 nt
43 nt
40 nt
37 nt
34 nt
31 nt
28 nt
25 nt
22 nt
6
7
8
9
10
39 nt
38 nt
41 nt
42 nt
43 nt
44 nt
THF
5'
3'
(CAG)3
(CAG)3
(GTC)3
GTC
(GTC)3
20 nt
49 nt
51 nt
54 nt
45 nt
THF
5'
3'
(CAG)3
(CAG)3
20 nt
(GTC)3
GTC
(GTC)3

Fig. S3

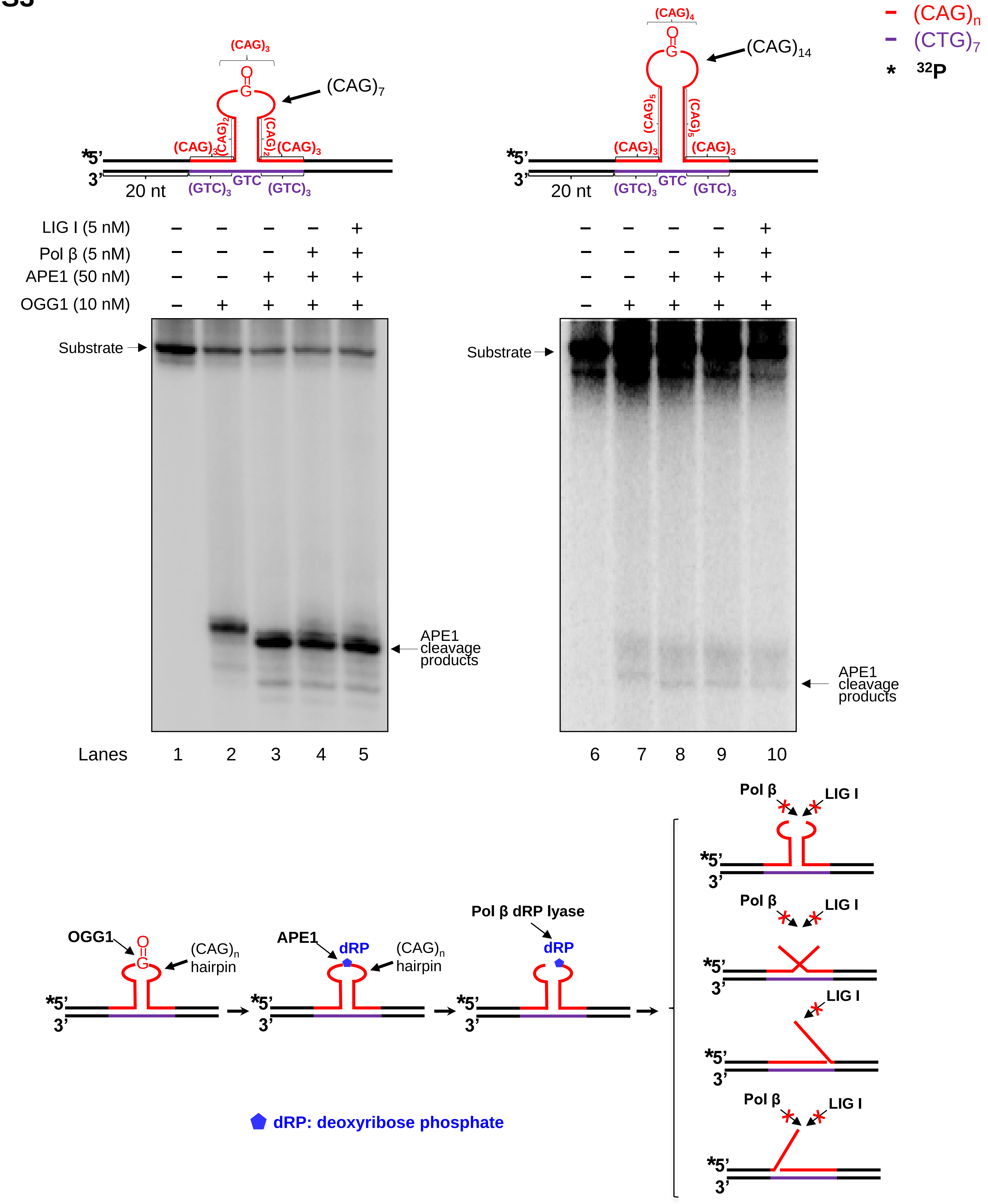

(CAG)n
(CTG)7
* 32P
(CAG)3
(CAG)7
(CAG)2
(CAG)3
*5'
3'
20 nt
(GTC)3
GTC
(CAG)4
(CAG)14
(CAG)5
LIG I (5 nM)
Pol β (5 nM)
APE1 (50 nM)
OGG1 (10 nM)
Substrate
APE1 cleavage products
Lanes
1
2
3
4
5
6
7
8
9
10
OGG1
(CAG)n hairpin
APE1
dRP
Pol β dRP lyase
Pol β
LIG I
dRP: deoxyribose phosphate

Fig. S4

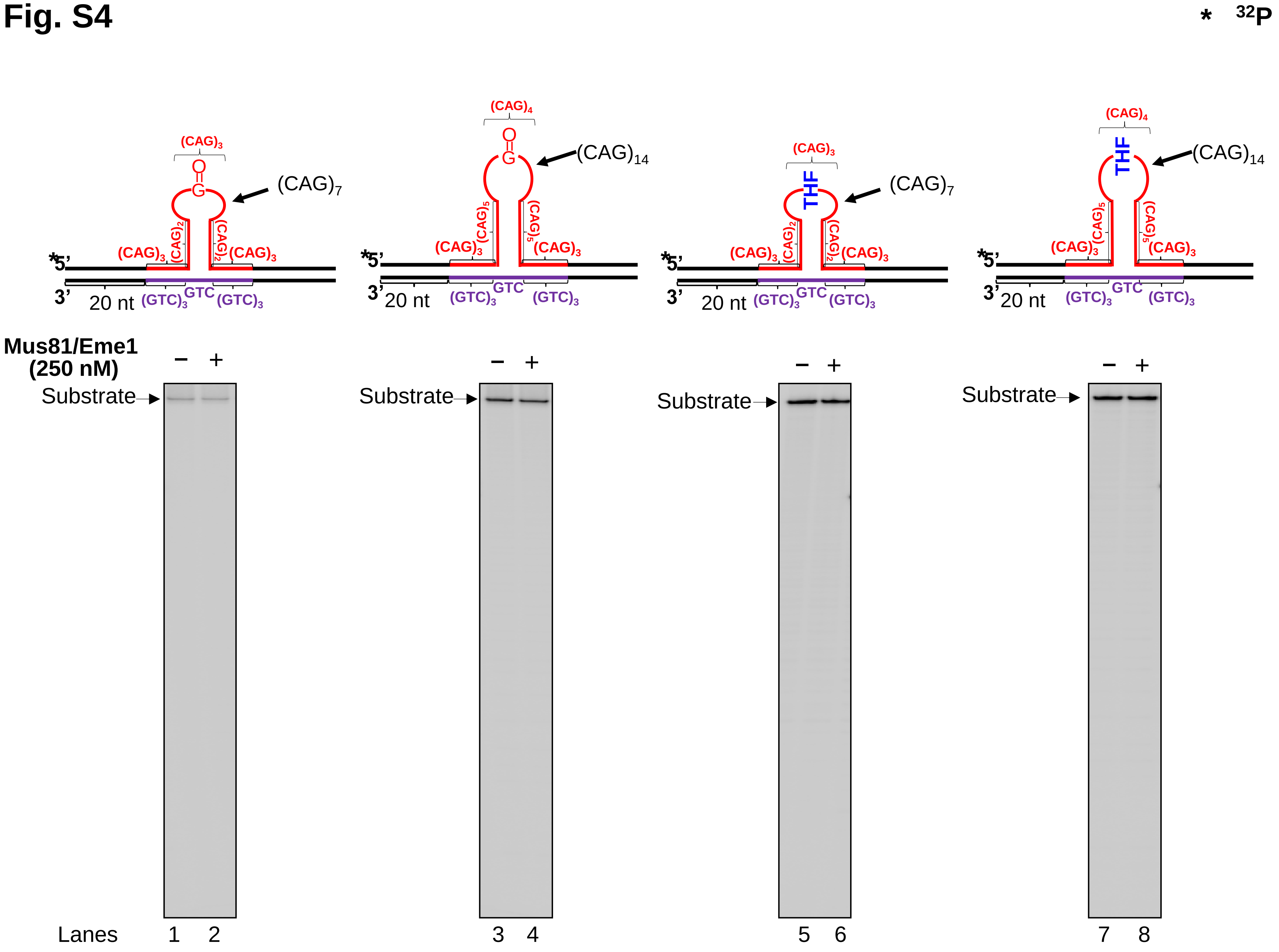
* 32P
(CAG)3
(CAG)7
(CAG)4
(CAG)14
THF
5'
3'
20 nt
(GTC)3
GTC
Mus81/Eme1 (250 nM)
− +
Substrate
Lanes
1 2
3 4
5 6
7 8

Fig. S5

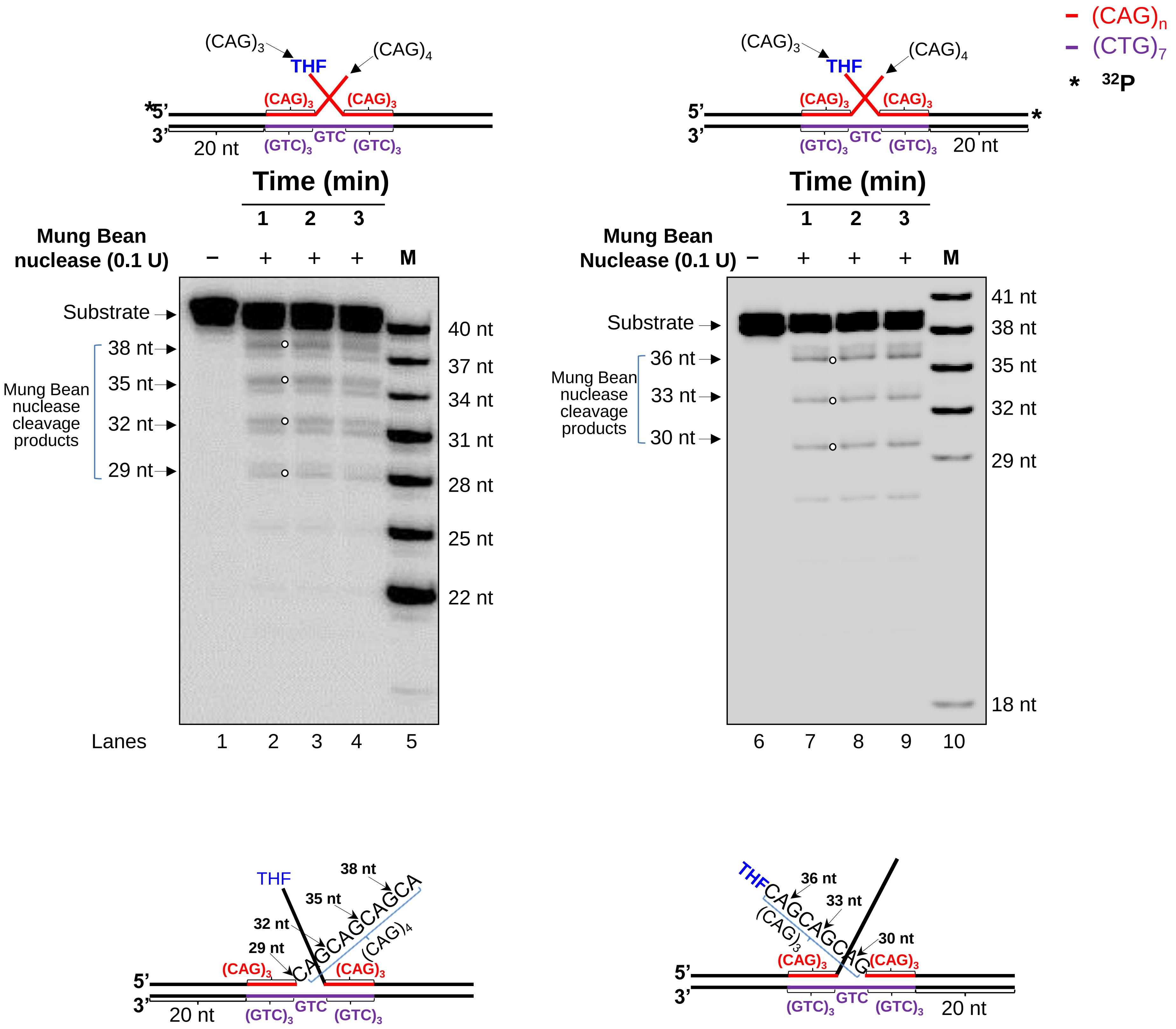
(CAG)n
(CTG)7
* 32P
(CAG)3
(CAG)4
THF
(CAG)3
(CAG)3
5'
3'
20 nt
(GTC)3
GTC
(GTC)3
Time (min)
1 2 3
Mung Bean nuclease (0.1 U)
− + + + M
Substrate
Mung Bean nuclease cleavage products
38 nt
35 nt
32 nt
29 nt
40 nt
37 nt
34 nt
31 nt
28 nt
25 nt
22 nt
Lanes
1 2 3 4 5
Mung Bean Nuclease (0.1 U)
36 nt
33 nt
30 nt
41 nt
38 nt
35 nt
32 nt
29 nt
18 nt
6 7 8 9 10
THF
CAGCAGCAGCA
(CAG)4
CAGCAGCAG
(CAG)3

Fig. S6

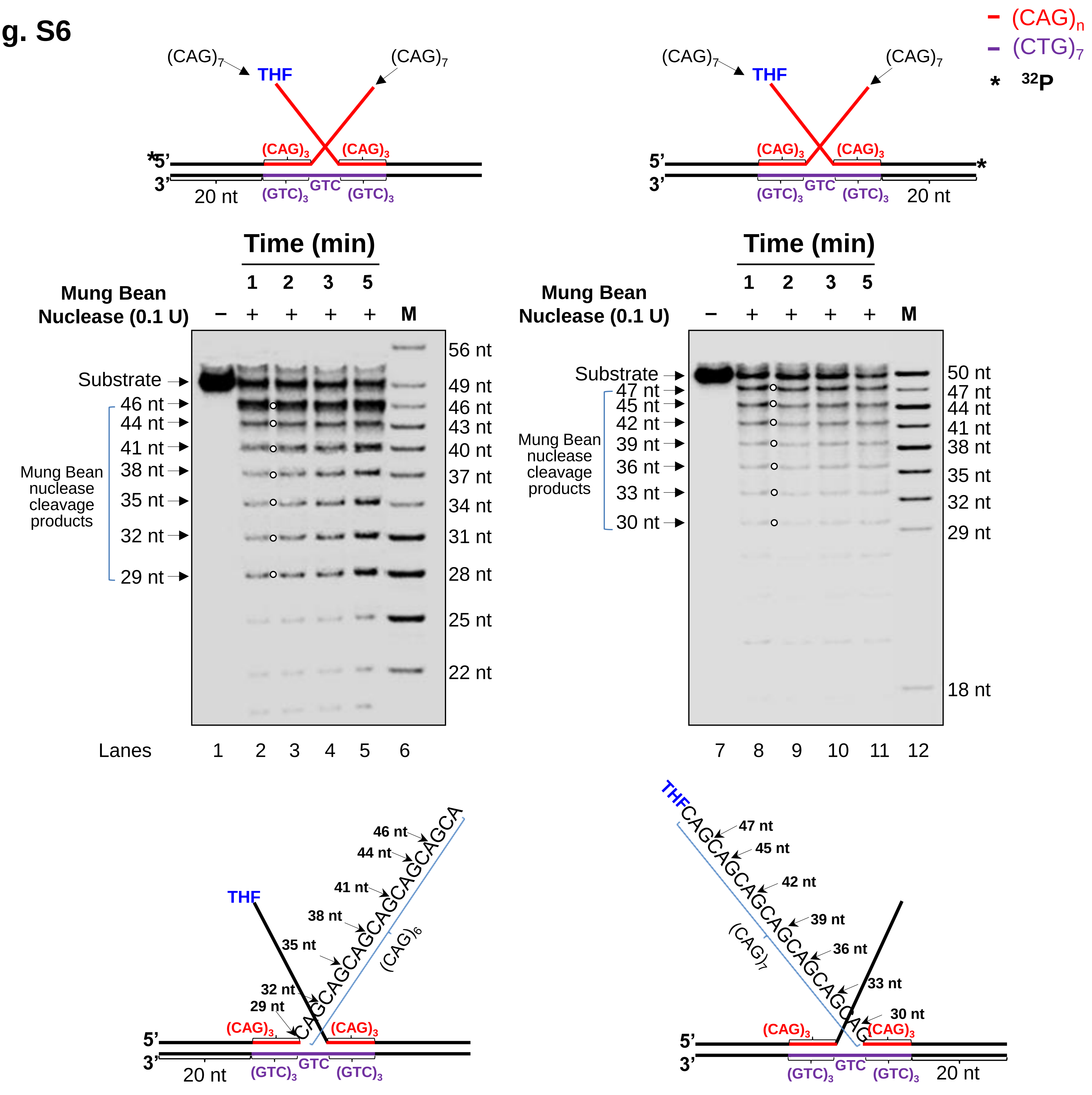

(CAG)n
(CTG)7
* 32P
(CAG)7
THF
(CAG)7
(CAG)3
(CAG)3
5'
3'
20 nt
(GTC)3
GTC
(GTC)3
Time (min)
1 2 3 5
Mung Bean Nuclease (0.1 U)
− + + + + M
Substrate
46 nt
44 nt
41 nt
38 nt
35 nt
32 nt
29 nt
Mung Bean nuclease cleavage products
56 nt
49 nt
46 nt
43 nt
40 nt
37 nt
34 nt
31 nt
28 nt
25 nt
22 nt
47 nt
45 nt
42 nt
39 nt
36 nt
33 nt
30 nt
50 nt
47 nt
44 nt
41 nt
38 nt
35 nt
32 nt
29 nt
18 nt
Lanes
1 2 3 4 5 6
7 8 9 10 11 12
CAGCAGCAGCAGCAGCAGCA
(CAG)6
(CAG)7
THFCAGCAGCAGCAGCAGCAGCAG

Fig. S7

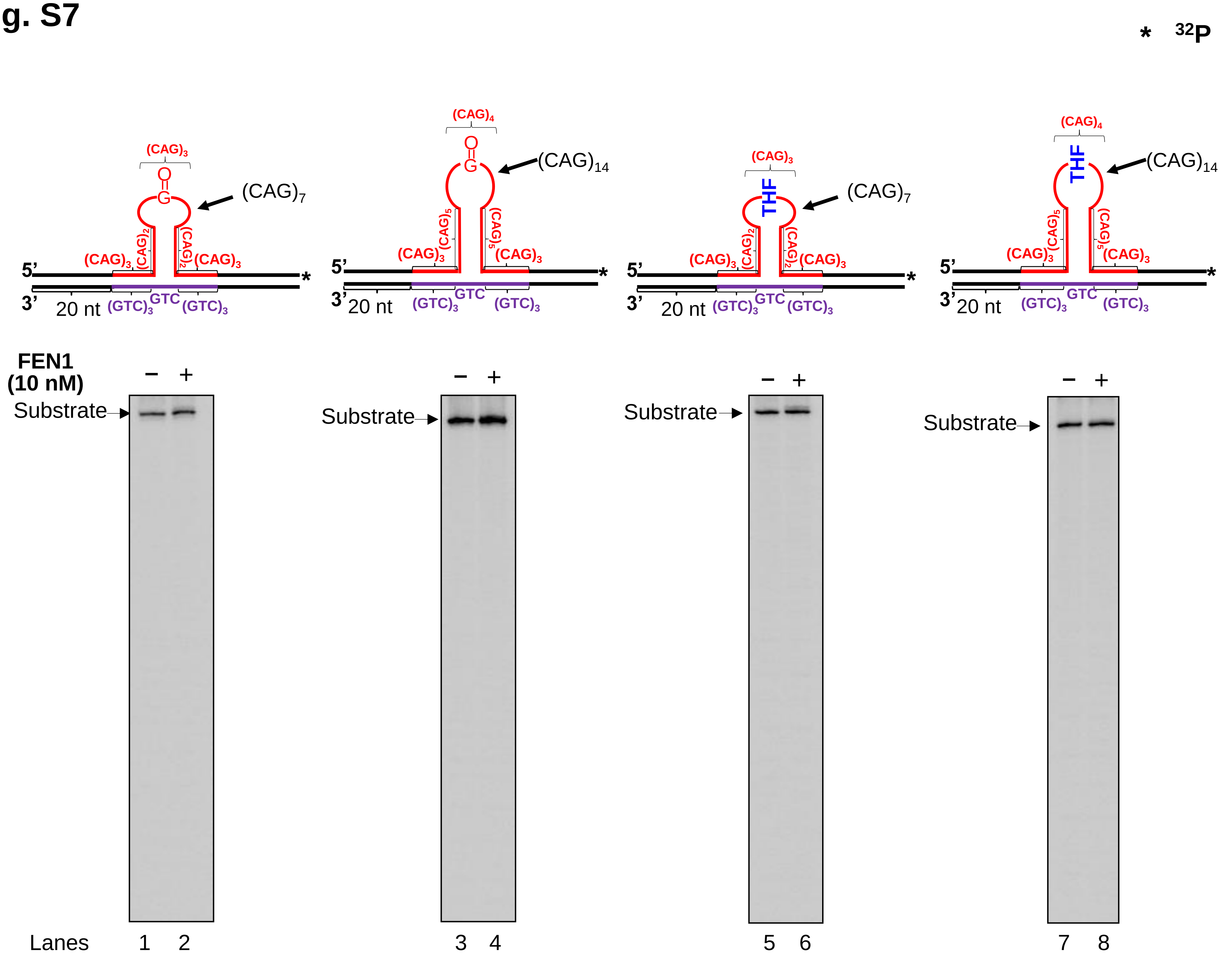
* 32P
(CAG)3
(CAG)7
(CAG)2
(CAG)3
5'
3'
20 nt
(GTC)3
GTC
(CAG)4
(CAG)14
(CAG)5
THF
FEN1
(10 nM)
− +
Substrate
Lanes
1 2
3 4
5 6
7 8

Supplementary Data

Supplementary Figure S1. Hairpin probing of $(CAG)_7$- and $(CAG)_{14}$-8-oxoG hairpin substrates

(A) The damaged strand of the $(CAG)_7$- or $(CAG)_{14}$-8-oxoG hairpin substrate was radiolabeled at the 3'-end. Substrates were incubated with 0.15 U of Mung Bean Nuclease at 1-, 2- and 3-min time intervals (lanes 2-4, and lanes 7-9). Lanes 1 and 6 represent the undigested substrate. Lanes 5 and 10 represent 3'-radiolabeled synthesized size markers (M) with 29 nt, 32 nt, 35 nt, 38 nt, 41 nt, 44 nt, 47 nt, 50 nt, 53 nt, 56 nt, 62 nt, 68 nt and 74 nt, respectively. (B) The damaged strand of the $(CAG)_7$- or $(CAG)_{14}$-8-oxoG substrates was radiolabeled at 5'-end. Substrates were incubated with 0.2 U or 0.5 U of S1 Nuclease at 3-, 5- and 10-min time intervals (lanes 2-4, and lanes 7-9). Lanes 1 and 6 represent the undigested substrate. Lanes 5 and 10 represent 5'-radiolabeled synthesized size markers (M) with 22 nt, 25 nt, 28 nt, 31 nt, 34 nt, 37 nt, 40 nt, 43 nt, 46 nt, 49 nt, 52 nt, 55 nt, 58 nt, 61 nt, 64 nt, 67 nt, and 70 nt, respectively. For all the experiments, 200 nM of substrate was used. Arrows and white dots indicate the major digestion products. The substrates are illustrated schematically above the gels. Hairpins deduced by a specific nuclease cleavage pattern and the nuclease digestion sites are illustrated schematically below the gels.

Supplementary Figure S2. Hairpin probing of $(CAG)_7$- and $(CAG)_{14}$-THF hairpin substrates

(A) The $(CAG)_7$- and $(CAG)_{14}$-THF hairpin substrates were radiolabeled at the 5'-end of the damaged strand. Substrates (200 nM) were incubated with 0.1 U of Mung Bean Nuclease at 1-, 2- and 3-min time intervals (lanes 2-4 and lanes 7−9). Lanes 1 and 6 represent the undigested substrate. Lanes 5 and 10 represent 5'-radiolabeled synthesized size markers (M) with 22 nt, 25 nt, 28 nt, 31 nt, 34 nt, 37 nt, 40 nt, 43 nt, 46 nt, 49 nt, 52 nt, 55 nt, 58 nt, 61 nt, 64 nt, 67 nt, and 70 nt, respectively. (B) 5'- radiolabeled substrates were incubated with 0.2 U or 0.5 U of S1 Nuclease at 3-, 5- and 10-min time intervals (lanes 2-4, and lanes 7-9). Lanes 1 and 6 represent the undigested substrate. Lanes 5 and 10 represent 5'-radiolabeled synthesized size markers (M) with 22 nt, 25 nt, 28 nt, 31 nt, 34 nt, 37 nt, 40 nt, 43 nt, 46 nt, 49 nt, 52 nt, 55 nt, 58 nt, 61 nt, 64 nt, 67 nt, and 70 nt, respectively. Arrows and white dots indicate the major nuclease digestion products. The substrates are illustrated schematically above the gels. Hairpins deduced by a specific nuclease cleavage pattern and the nuclease digestion sites are illustrated schematically below the gels.

Supplementary Figure S3. Short-patch BER of $(CAG)_7$- and $(CAG)_{14}$-8-oxoG hairpin

containing substrates

Short-patch BER of an 8-oxoG in the loop region of a CAG hairpin was examined by incubating the $(CAG)_7$- and $(CAG)_{14}$-8-oxoG substrates (25 nM) with OGG1 (10 nM) alone (lanes 2 and 7), or OGG1 (10 nM) along with APE1 (50 nM) (lanes 3 and 8) or OGG1 (10 nM) along with both APE1 (50 nM) and pol β (5 nM) (lanes 4 and 9) or OGG1 (10 nM) along with APE1 (50 nM), pol β (5 nM) and LIG (5 nM)(lanes 5 and 10) at 37°C for 30 min. Lanes 1 and 6 correspond to substrates only. Substrates were ^{32}P-labeled at the 5'-end of their damaged strands and are illustrated schematically above the gels. The scheme of short-patch BER of an 8-oxoG in the hairpin substrates is illustrated below the gels.

Supplementary Figure S4. Mus81/Eme1 cleavage of a hairpin

The $(CAG)_7$-8-oxoG/THF and $(CAG)_{14}$-8-oxoG/THF substrates were radiolabeled at the 5'-end of the hairpin containing strand. Substrates (25 nM) were incubated with 250 nM purified Mus81/Eme1 at 37°C for 30 min (lanes 2, 4, 6 and 8). Lanes 1, 3, 5 and 7 represent substrate alone. Substrates are illustrated schematically above the gels.

Supplementary Figure S5. Flap probing of $(CAG)_3$/$(CAG)_4$ double-flap substrate

A substrate with a 3'-$(CAG)_4$ flap and a 5'-$(CAG)_3$-THF flap was radiolabeled at the 5'-end (left panel) of the upstream strand or the 3'-end (right panel) of the downstream strand for probing the formation of an upstream 3'-flap and a downstream 5'-flap. The substrate (200 nM) was incubated with 0.1 U of Mung Bean Nuclease at 1-, 2- and 3-min time intervals (lanes 2-4 and lanes 7-9). Lanes 1 and 6 represent an undigested substrate. Lanes 5 and 10 represent 5'-radiolabeled synthesized size markers (M) with 22 nt, 25 nt, 28 nt, 31 nt, 34 nt, 37 nt, and 40 nt, respectively (left panel) and 3'-radiolabeled size markers (M) with 18 nt, 29 nt, 32 nt, 35 nt, 38 nt, and 41 nt, respectively. Arrows and white dots indicate the major nuclease digestion products. The substrates are illustrated schematically above the gels. Double-flaps deduced by a specific nuclease cleavage pattern and the nuclease digestion sites are illustrated schematically below the gels.

Supplementary Figure S6. Flap probing of $(CAG)_7$ double-flap substrate

A substrate with a 3'-$(CAG)_7$ - and 5'-$(CAG)_7$ -THF flap was radiolabeled at the 5'-end of the upstream, strand (left panel) or the 3'-end of the downstream strand (right panel). The substrate (200 nM) was incubated with 0.1 U of Mung Bean Nuclease at 1-, 2-, 3-, and 5-min time intervals (lanes 2-5 and lanes 8-11). Lanes 1 and 7 represent an undigested substrate. Lane 6 represents 5'-

radiolabeled synthesized size markers (M) with 22 nt, 25 nt, 28 nt, 31 nt, 34 nt, 37 nt, 40 nt, 43 nt, 46 nt, 49 nt and 56 nt for probing the formation of a 3'-flap. Lane 12 represents 3'-radiolabed size markers (M) with 18 nt, 29 nt, 32 nt, 35 nt, 38 nt, 41 nt, 44 nt, 47 nt and 50 nt for probing the formation of a 5'-flap. Arrows and white dots indicate the major nuclease digestion products. Substrates are illustrated schematically above the gels. Double-flaps deduced by a specific nuclease cleavage pattern and the nuclease digestion sites are illustrated schematically below the gels.

Supplementary Figure S7. FEN1 cleavage of a hairpin

The $(CAG)_7$-8-oxoG/THF and $(CAG)_{14}$-8-oxoG/THF hairpin substrates were labeled at the 3'-end of the hairpin-containing strand. Substrates (25 nM) were incubated with 10 nM purified FEN1 at 37°C for 30 min (lanes 2, 4, 6 and 8). Lanes 1, 3, 5 and 7 represent a substrate alone. Substrates are illustrated schematically above the gels.
